# Transcriptomic comparison between beetle strains selected for short and long durations of death feigning

**DOI:** 10.1038/s41598-019-50440-5

**Published:** 2019-09-30

**Authors:** Hironobu Uchiyama, Ken Sasaki, Shogo Hinosawa, Keisuke Tanaka, Kentarou Matsumura, Shunsuke Yajima, Takahisa Miyatake

**Affiliations:** 1 NODAI Genome Research Center, Tokyo University of Agriculture, 1-1-1 Sakuragaoka, Setagaya-ku, Tokyo 156-8502 Japan; 2 Department of Bioscience, Tokyo University of Agriculture, 1-1-1 Sakuragaoka, Setagaya-ku, Tokyo 156-8502 Japan; 3 Graduate School of Agriculture, Tamagawa University, Machida, Tokyo 194-8610 Japan; 4 Graduate School of Environmental and Life Science, Okayama University, 1-1-1 Tsushina-naka, Kita-ku, Okayama 700-8530 Japan

**Keywords:** Ecology, Behavioural ecology

## Abstract

The molecular basis of death feigning, an antipredator behavior that has received much attention recently, was analyzed. We compared the gene expression profiles of strains with different behaviors, i.e., different durations of death feigning, in the beetle *Tribolium castaneum*. Beetles artificially selected for short (S) and long (L) durations of death feigning for many generations were compared thoroughly by RNA sequencing. We identified 518 differentially expressed genes (DEGs) between the strains. The strains also showed divergence in unexpected gene expression regions. As expected from previous physiological studies, genes associated with the metabolic pathways of tyrosine, a precursor of dopamine, were differentially expressed between the S and L strains; these enzyme-encoding genes were expressed at higher levels in the L strain than in the S strain. We also found that several genes associated with insulin signaling were expressed at higher levels in the S strain than in the L strain. Quantitative real-time PCR analysis showed that the relative expression levels of *Tchpd* (encoding 4-hydroxyphenylpyruvate dioxygenase, *Hpd*) and *Tcnat* (encoding N*-acetyltransferase*, *Nat*) were significantly higher in the L strain than in the S strain, suggesting the influence of these enzymes on the supply of dopamine and duration of death feigning.

## Introduction

Since Edmunds^1^, much research has focused on the behaviors adopted by animals to avoid attack by enemies^2^. To date, several molecular analyses have been conducted on traits associated with anti-predator behaviors^3,4^. However, to our knowledge, there have been few analyses of gene expression associated with death feigning as an animal defense behavior. Many animal species escape from enemies by pretending to be dead, i.e., death feigning or tonic immobility^1,2,5–8^. The red flour beetle, *Tribolium castaneum*, used in the present study feigns death when attacked by its natural enemy, the jumping spider^5,9,10^. In group-living beetles, individuals that adopt tonic immobility can survive by sacrificing neighbors that is, the immobility following a spider attack is selfish; death-feigning prey increase their probability of survival at the expense of their mobile neighbors^10^. Therefore, death feigners can survive as a minor group in the wild.

In the laboratory, strains divergently selected for short (S strains) and long (L strains) durations of death feigning, which is activated by external stimuli, have been established^5,9^. When a trio, consisting of an adult beetle from each of the S and L strains and a jumping spider as predator, was placed in an arena, the S strain beetle never survived, while the L strain beetle survived in all the cases (N = 19) tested^10^.

The evolution of gene expression and gene interactions under the selective environment is unknown. Therefore, which and how many genes are differentially expressed in the bodies of the beetles between the strains, and which beetle did or did not survive the predator attack, are rather interesting questions. Previous studies have revealed that S strain beetles move actively on a routine basis, while L strain beetles do not move much^9^. Significantly higher expression of dopamine was observed in the brain of the S strain beetle than in that of the L strains^9^. It is also known that caffeine, which activates dopamine signaling, shortened the duration of immobility of L strain beetles^11^. These findings were evidence of the effectiveness of dopamine in the escape from enemies and duration of death feigning in *T*. *castaneum*. Another study showed a difference in stress tolerance between the strains: L strain beetles exhibited weaker responses to heat, cold, and vibration stress than S strain beetles^12^. Based on these results, we expect differences between the S and L strains of *T*. *castaneum* in many parts of the gene expression pathways.

Hence, we compared the gene expressions of the S and L strains exhaustively by RNA sequencing (RNA-seq). We identified differentially expressed genes (DEGs) between strains, characterized these genes by gene ontology (GO) analysis and examined the metabolic pathways of the significant functional genes.

## Results

### Comparative gene expression analysis

An RNA-seq analysis was performed to compare the gene expression levels of the strains. Approximately 24–30 million raw reads were generated from cDNA libraries using the HiSeq 2500 platform (S1 Table). After sequence trimming and quality filtering, approximately 90% of the raw reads remained in each sample. In addition, approximately 90% of the clean reads were mapped to the Tcas5.2 reference genome (https://metazoa.ensembl.org/Tribolium_castaneum/Info/Annotation/).

**Figure 1:**
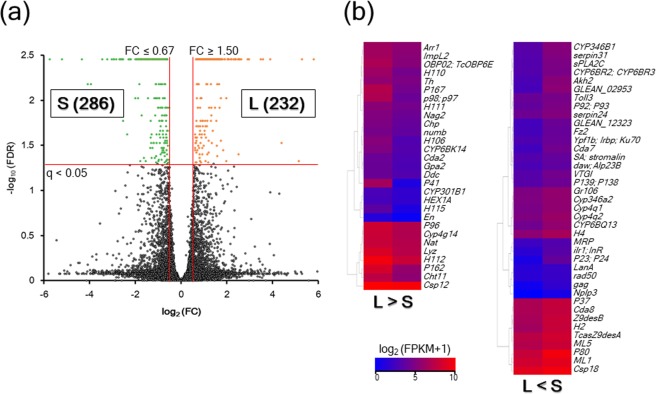
Volcano plot (**a**) and heatmap (**b**) for comparisons between the L and S strains. (**a**) Volcano plot showing log2 FC (x-axis) and log10 FDR corrected *P*-value (y-axis) points of all the expressed genes in the Tcas5.2 reference genome using Microsoft Office Excel. The orange dots represent 232 DEGs of the L strain (FC ≥ 1.5 and q < 0.05), whereas the green dots represent 286 DEGs of the S strain (FC ≤ 0.67 and q < 0.05). (**b**) A heatmap showing 68 DEGs with defined locus names was created using the R package “gplots”. The color gradient represents high-expression (red) to low-expression (blue) by calculating log2 (FPKM + 1).

A total of 518 DEGs were detected in a comparison of the L and S strains (S2 Table). Of these DEGs, significant upregulation in the L strain was observed for 232 DEGs, whereas upregulation in the S strain was indicated for 286 DEGs (Fig. 1A). Furthermore, 68 DEGs with defined locus names are shown on a heatmap (Fig. 1B). In these DEGs, the genes upregulated in the L strain (Arr1, Cda2, Chp, Cht11, Csp12, CYP301B1, Cyp4g14, CYP6BK14, Ddc, En, Gpa2, H106, H110, H111, H112, H115, HEX1A, ImpL2, Lyz, Nag2, Nat, numb, OBP02, TcOBP6E, P162, P167, P41, P96, p98; p97, and Th) were more numerous than those upregulated in the S strain (CYP346B1, serpin31, sPLA2C, CYP6BR2, CYP6BR3, Akh2, GLEAN_02953, Toll3, P92, P93, serpin24, GLEAN_12323, Fz2, Ypf1b, Irbp, Ku70, Cda7, SA, stromalin, daw, Alp23B, VTGl, P139, P138, Gr106, Cyp346a2, Cyp4q1, Cyp4q2, CYP6BQ13, H4, MRP, ilr1, InR, P23, P24, LanA, rad50, gag, Nplp3, P37, Cda8, Z9desB, H2, TcasZ9desA, ML5, P80, ML1, and Csp18).

### Enrichment analysis for DEGs

Using the Database for Annotation, Visualization and Integrated Discovery (DAVID) 6.8 program, the DEGs of each strain were enriched in GO terms under biological process (BP), cellular component (CC), and molecular function (MF) and examined against the Kyoto Encyclopedia of Genes and Genomes (KEGG) pathway database^13–15^ (Table 1). The L strain was significantly enriched in seven BPs (“aromatic amino acid family metabolic process”, “visual perception”, “carbohydrate metabolic process”, “chitin metabolic process”, “biosynthetic process”, “phototransduction”, and “lipid catabolic process”), one CC (“extracellular region”), five MFs (“pyridoxal phosphate binding”, “beta-N-acetylhexosaminidase activity”, “serine-type endopeptidase activity”, “substrate-specific transmembrane transporter activity”, and “chitin binding”), and nine KEGG pathways (“lysosome”, “phenylalanine metabolism”, “glycosphingolipid biosynthesis - globo series”, “other glycan degradation”, “metabolic pathways”, “tyrosine metabolism”, “glycosphingolipid biosynthesis - ganglio series”, “amino sugar and nucleotide sugar metabolism”, and “glycosaminoglycan degradation”). On the other hand, the S strain was enriched significantly in one BP (“biosynthetic process”), five MFs (“monooxygenase activity”, “serine-type endopeptidase activity”, “oxidoreductase activity, acting on paired donors, with incorporation or reduction of molecular oxygen”, “oxidoreductase activity, acting on paired donors, with oxidation of a pair of donors resulting in the reduction of molecular oxygen to two molecules of water”, and “transporter activity”), and two KEGG pathways (“fatty acid metabolism” and “one carbon pool by folate”).

**Table 1:** Significantly enriched gene ontology and pathway terms for DEGs of the L and S strains.

DEGs	Category	ID	Term	P-value	Fold enrichment
L strain	GO_BP	GO:0009072	Aromatic amino acid family metabolic process	9.11E-05	38.08333333
		GO:0007601	Visual perception	0.001688392	42.84375
		GO:0005975	Carbohydrate metabolic process	0.008177236	3.808333333
		GO:0006030	Chitin metabolic process	0.009132668	5.829081633
		GO:0009058	Biosynthetic process	0.033210108	10.08088235
		GO:0007602	Phototransduction	0.033993886	57.125
		GO:0016042	Lipid catabolic process	0.044916479	8.56875
	GO_CC	GO:0005576	Extracellular region	0.00719112	4.724358974
	GO_MF	GO:0030170	Pyridoxal phosphate binding	0.001040993	10.64
		GO:0004563	Beta-N-acetylhexosaminidase activity	0.004872917	26.6
		GO:0004252	Serine-type endopeptidase activity	0.006847854	4.047826087
		GO:0022891	Substrate-specific transmembrane transporter activity	0.014494756	7.6
		GO:0008061	Chitin binding	0.019565587	4.75
	KEGG pathway	tca04142	Lysosome	1.68E-04	4.653095844
		tca00360	Phenylalanine metabolism	5.81E-04	22.16565657
		tca00603	Glycosphingolipid biosynthesis - globo series	9.85E-04	18.75555556
		tca00511	Other glycan degradation	0.001680184	9.235690236
		tca01100	Metabolic pathways	0.001965983	1.706482518
		tca00350	Tyrosine metabolism	0.002239084	14.34248366
		tca00604	Glycosphingolipid biosynthesis - ganglio series	0.006623679	22.85833333
		tca00520	Amino sugar and nucleotide sugar metabolism	0.015469369	4.996357013
		tca00531	Glycosaminoglycan degradation	0.039794975	9.143333333
S strain	GO_BP	GO:0009058	Biosynthetic process	0.045794874	8.489164087
	GO_MF	GO:0004497	Monooxygenase activity	0.007051684	4.028764159
		GO:0004252	Serine-type endopeptidase activity	0.012864537	3.547064097
		GO:0016705	Oxidoreductase activity, acting on paired donors, with incorporation or reduction of molecular oxygen	0.029318076	3.411113905
		GO:0016717	Oxidoreductase activity, acting on paired donors, with oxidation of a pair of donors resulting in the reduction of molecular oxygen to two molecules of water	0.034384389	9.989690722
		GO:0005215	Transporter activity	0.048287502	3.586042823
	KEGG pathway	tca01212	Fatty acid metabolism	7.36E-04	6.130587484
		tca00670	One carbon pool by folate	0.024502313	11.82327586

From these metabolic and signaling pathways, we focused on three groups of genes: tyrosine metabolic pathways, insulin-related genes and longevity genes (Table 2). In tyrosine metabolism, all five enzyme-encoding genes were DEGs of the L strain, whereas all five genes involved in insulin secretion and signaling and four of the five genes involved in longevity were DEGs of the S strain (Table 2).

**Table 2:** List of differentially expressed genes in tyrosine metabolic pathways, insulin related genes and longevity-related genes by comparison between L and S lines.

LOC name	Description	log2(fold change + 1)	P-value	FDR
**Tyrosine metabolic pathways**				
Th	Tyrosine hydroxylase (TH)	0.776900946	5.00E-05	0.00352415
Ddc	Dopa decarboxylase (DDC)	0.894929643	5.00E-05	0.00352415
Nat	Dopamine N acetyltransferase (NAT)	0.677680435	0.00055	0.0281853
LOC659321	Tyrosine aminotransferase (TAT)	0.644737903	0.0005	0.0259129
LOC662658	4-Hydroxyphenylpyruvate dioxygenase (HPD)	1.020744899	5.00E-05	0.00352415
**Insulin- related genes**				
ilr1; InR	Insulin-like receptor (INSR)	−1.639107707	0.00095	0.0436105
LOC660178	Fatty acid synthase (FASN)	−0.510937173	0.0002	0.0120247
LOC661092	Sodium/potassium-transporting ATPase subunit alpha (ATP1A)	−0.830887715	5.00E-05	0.00352415
LOC103312937	Sodium/potassium-transporting ATPase subunit beta-2 (ATP1B)	−0.868987214	5.00E-05	0.00352415
Gr106	Glucose transporter (GLUT)	−0.755722844	0.0007	0.0338289
**Longevity- related genes**				
LOC662610	Putative fatty acyl-CoA reductase CG5065 (FAR)	−1.884937074	0.00015	0.00949185
LOC657196	Protein lethal(2)essential for life (CRYAB)	−1.014980659	5.00E-05	0.00352415
TcasZ9desA	Z9 acyl-CoA desaturase A (SCD)	−0.823645486	0.00075	0.0357178
LOC662326	Heat shock 70 kDa protein cognate 2 (HSPA)	−0.652629284	0.0002	0.0120247
LOC656567	Glutamate–cysteine ligase catalytic subunit (GCLC)	0.735412263	5.00E-05	0.00352415

### Tyrosine metabolic pathway analysis

**Figure 2:**
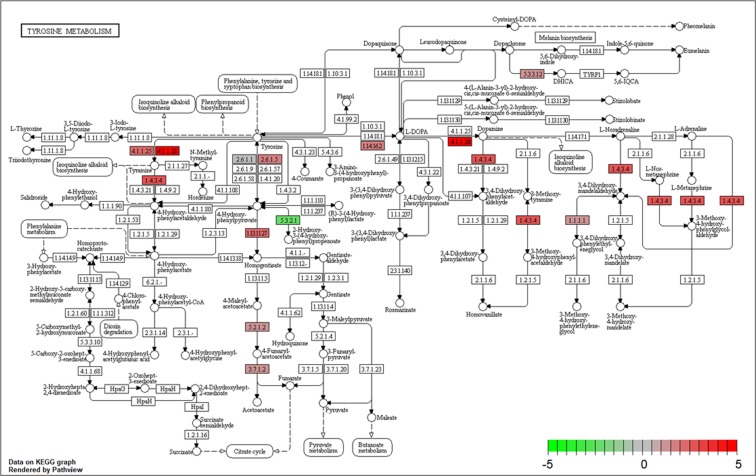
KEGG pathway for tyrosine metabolism in *T*. *castaneum* (tca00350). The color gradient represents differences in the intensity of FC between the L and S strains. The FC was calculated as (FPKM of the L strain + 0.1)/(FPKM of the S strain + 0.1) when the expression value of the L strain was higher than that of the S strain. In contrast, FC was calculated as (FPKM of the S strain + 0.1)/(FPKM of the L strain + 0.1) × (−1) when the expression value of the L strain was lower than that of the S strain.

The association of tyrosine metabolism with dopamine biosynthesis was identified in the above enrichment analysis. Here, we explore this aspect via pathway analysis (Fig. 2). Three related enzymes tended to be more highly activated in the L strain than in the S strain: aromatic -L-amino-acid decarboxylase (Ddc) (EC: 4.1.1.28), which is involved in the biosynthesis of tyramine and dopamine, and tyrosine aminotransferase (Tat) (Ec: 2.6.1.5) and 4-hydroxyphenylpyruvate dioxygenase (Hpd) (EC: 1.13.11.27), which are involved in the biosynthesis of homogentisate from tyrosine. Genes encoding these enzymes were found among the DEGs of the L strain (Table 2). Monoamine oxidase (EC: 1.4.3.4) is involved in the biosynthesis of 4-hydroxyphenylacetaldehyde, 3,4-dihydroxyphenylacetaldehyde, 3-methoxytyramine, 3-methoxy-4-hydroxyphenylacetaldehyde, 3,4-dihydroxymandelaldehyde, and 3-methoxy-4-hydroxyphenylglycolaldehyde, although the gene encoding this enzyme was not included among the DEGs (Table S2). Genes encoding these enzymes were included in the DEGs of the L strain (S2 Table). The gene expression of *Nat*, encoding dopamine N-acetyltransferase, was also higher in the L strain than in the S strain (Fig. 1B and Table 2), although this gene was not included in the tyrosine metabolism in the pathway enrichment analysis (Fig. 2).

### Quantitative real-time PCR (qPCR) analyses

**Figure 3:**
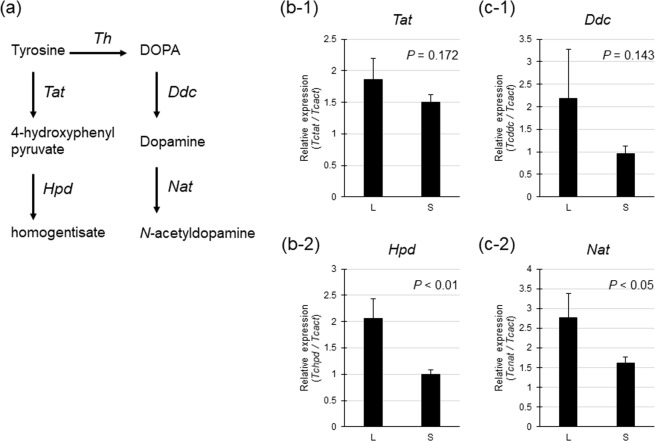
Relative expression of genes involved in tyrosine metabolism and dopamine metabolism in the L and S strains. a: Metabolic pathways involving the conversion of tyrosine to homogentisate and *N*-acetyldopamine in insects. Ddc: DOPA decarboxylase, Hpd: 4-hydroxyphenylpyruvate dioxygenase, Nat: *N*-acetyltransferase, Tat: tyrosine aminotransferase, Th: tyrosine hydroxylase. b: Relative gene expression of *Tcddc* and *Tcnat*, as determined by qPCR. c: Relative gene expression of *Tcddc* and *Tcnat*, as determined by qPCR. Groups were compared between the L and S strains by the t-test.

In tyrosine metabolism in insects (Fig. 3A), the relative expression levels of *Tctat* (encoding the Tat enzyme) were not significantly different between the L and S strains (*t*-test, *t* = 0.972, *P* = 0.172, N_L_ = N_S_ = 10, Fig. 3B-1), whereas those of *Tchpd* (encoding the Hpd enzyme) were significantly higher in the L than in the S strain (*t* = 2.771, *P* < 0.01, N_L_ = N_S_ = 10, Fig. 3B-2). In dopamine metabolism, the relative expression of *Tcddc* (encoding the Ddc enzyme) did not differ between the L and S strains (t-test, t = 1.101, *P* = 0.143, N_L_ = N_S_ = 10, Fig. 3C-1), whereas that of *Tcnat* (encoding the Nat enzyme) was significantly higher in the L strain than in the S strain (*t* = 1.821, *P* < 0.05, N_L_ = N_S_ = 10, Fig. 3C-2).

## Discussion

Behavioral mutations based on genetic factors are thought to be caused by gene expression associated with neural properties and accompany the expression of many genes involved in cell signaling cascades. The present study demonstrated that two behavioral strains that had been selected artificially for short and long durations of death feigning differentially expressed a group of genes involved in neural metabolic pathways, stress-response, and nutrient-sensing. Some of these genes have also been indicated by previous physiological studies, and the results are consistent with physiological data.

### Tyrosine metabolic pathways

Five genes in tyrosine metabolic pathways were expressed differentially between the strains (Table 2). Tyrosine is a precursor of dopamine and is also an important component of peptides and proteins. Therefore, tyrosine can be metabolized into several pathways. Our results showed that three different metabolic pathways of tyrosine were upregulated in the individuals of the L strain (Figs 2 and 3A). One pathway was dopamine metabolism, involving the conversion of tyrosine to *N*-acetyldopamine via dopamine. This pathway includes Th (tyrosine to DOPA), Ddc (DOPA to dopamine), and Nat (dopamine to *N-*acetyldopamine). The second pathway involved the metabolism of tyrosine to homogentisate. This pathway includes Tat (tyrosine to 4-hydroxyphenil) and Hpd (4-hydroxyphenyl to homogentisate). The third pathway was tyramine metabolism to 4-hydroxyphenylacetaldehyde. This pathway includes aromatic L-amino-acid decarboxylase or Tdc (tyrosine to tyramine) and Mao (tyramine to 4-hydroxyphenylacetldehyde).

Dopamine synthesis is mediated by *Th* and *Ddc*, while dopamine metabolism is mediated by *Nat*. According to the RNA-seq results, the three enzyme-encoding genes were expressed at higher levels in the L strain than in the S strain. The results of the qPCR analyses supported the high expression of the *Nat* gene (*Tcnat*) in the L strain. Tyrosine metabolism is mediated by *Tat* and *Hpd*. The results of RNA-seq indicated that the genes encoding these enzymes were expressed at higher levels in the L than in the S strain (Fig. 2 and Table 2). The results of qPCR analyses supported the high expression of the Hpd gene (*Tchpd*) in the L strain (Fig. 3B-2). It has been reported that individuals of the L strain have lower dopamine levels in the head than those of the S strain, which is associated with the promotion of locomotor activities and the short duration of death feigning in the S strain^9,11^. In the L strain, the metabolism of tyrosine, a precursor of dopamine, is active, and tyrosine metabolism activity is high outside the dopamine synthesis pathway. As a result, large amounts of tyrosine are metabolized in the L strain, and thus, the entry of tyrosine into the dopamine synthesis pathway may be less than that in the S strain. On the other hand, the expression of *Nat*, which degrades dopamine, is high in the L strains, and thus, there is a possibility that greater amounts of dopamine are degraded in the L strain than in the S strain. Tyrosine may be metabolized actively by three different pathways in the L strain, and dopamine may also be converted actively into several metabolites, including *N*-acetyldopamine, in the L strain (see Fig. 2). Such active conversion of tyrosine and dopamine in multiple pathways in the L strain might explain the low dopamine levels in the L strain.

Dopamine-related substances, including DOPA, dopamine and *N*-acetyldopamine, can be substrates for melanin synthesis and sclerotization of the cuticle and promote insect defense systems^13,14^. The upregulation of Th, Ddc, and Nat in the L strain has the potential to promote the nonneuronal activities that regulate dopamine-related substances.

### Stress-responsive genes

Five genes associated with longevity were expressed differentially between the strains (Table 2). Antioxidant material enzyme-encoding genes are associated with oxygen metabolism, and the expression of these genes increases when an animal is under stress. The enzyme system uses antioxidant enzyme-encoding genes, such as *Cat* (catalase) and *Gbp* (growth-blocking peptide), that are indispensable to counteract the effect of antioxidant material enzyme-encoding genes. A previous study revealed that beetles of the L strain are highly sensitive to environmental stress such as mechanical vibration and high or low temperatures^12^. Therefore, stress-responsive genes such as heat shock proteins (*Hsps*), superoxide dismutase (*Sod*), glutathione peroxidase (*Gpx*), and catalase (*Cat*) of S and L strains were compared, but beetles derived from the S strain exhibited high expression of only catalase compared to those of the L strain^12^. In the present study (see Table 2), genes associated with heat shock proteins (HSPs, LOC662326: heat shock 70 kDa protein cognate 2) and glutathione (LOC657602: glutathione S-transferase) were differentially expressed in the S and L strains.

### Insulin signaling pathways

Five insulin-related genes were expressed differentially between the strains (Table 2). Insulin signaling pathways are nutrient-sensing and growth-regulating signaling cascades that affect polymorphisms within a species and affect life history and longevity^15^. The upregulation of genes associated with insulin signaling pathways and insulin secretion generally prolongs longevity in insects^16,17^. In our results, several insulin signaling-related genes were expressed more highly in the S strain than in the L strain (Table 2). These results predict prolongation of longevity in the S strain. As described above, the survival rates were lower in the L strain than in the S strain under some stress conditions^12^. However, survival rates under natural conditions were not compared between the strains because of the substantial longevity. Therefore, it is necessary to examine the longevity of both strains in the future.

Insulin-signaling pathways also regulate activities associated with tyrosine metabolism in *Drosophila melanogaster* females^18^. Mutation of the insulin-like peptide DILP6 (*dilp6*) results in an increase in the activities of tyrosine metabolic enzymes (alkaline phosphatase and Th). The opposite relationship between insulin signaling and tyrosine metabolism to *D*. *melanogaster* was found in the *T*. *castaneum* strains that exhibited death feigning. Our results showed relatively low gene expression of insulin signaling-related genes with high expression of tyrosine metabolic genes, including *Th*, in the L strain. These results highlight a link between the insulin-signaling pathway and dopamine and death feigning.

Furthermore, there is a possibility that the other genes that were expressed differentially in the S and L strains may also be associated with the duration of death feigning as side effects. Functional validation of the genes that became candidates in the present transcriptome analysis to relate the duration of death-feigning behavior are required in the future.

## Conclusion

Strains selected divergently for short and long durations of death feigning showed unexpected divergence in several gene expression regions. As expected from previous neurobiological studies^9,11,19^, genes associated with metabolic pathways of tyrosine, a precursor of dopamine, exhibited differential expression between the S and L strains; these enzyme-encoding genes were expressed at higher levels in the L strain than in the S strain. We also found that several insulin signaling-related genes were expressed at higher levels in the S strain than in the L strain. Although previous experiments showed that beetles derived from the S strain had high gene expression levels of only catalase compared to the L strain^12^, there were no differences in the expression of stress-responsive genes. In the future, analyses of these genes are required.

## Materials and Methods

### Insects

The red flour beetle, *Tribolium castaneum* (Herbst, 1797), is a stored-product insect found worldwide and a model genome species^20^. The protocol for artificial selection for the duration of death feigning was described in Miyatake *et al*.^5^. Briefly, the duration of death feigning was measured in 100 male and 100 female adult beetles that were randomly selected. From the population, 10 males and 10 females with the shortest and longest durations of death feigning were allowed to reproduce for the next generation. The selection regime was continued for more than 20 generations^9^.

### RNA extraction and cDNA library preparation

We used only female beetles (adults one month after emergence) for RNA-seq because we wanted to eliminate the influence of sex. Ten female individuals of the S or L strains, at one beetle per sample, were placed in an Epplendorf tube, and liquid nitrogen was placed in each tube. Abdomens and legs were removed from the frozen bodies using a pair of fine spring scissors on a cooling plate under a stereomicroscope as the abdomen sometimes include items consumed by the beetle or microorganisms. We used the head and thoracic parts for RNA isolation. Before RNA extraction, we washed the head and thoracic parts with sterile water. RNA was extracted from the tissue using an RNA isolation kit: NucleoSpin (Macherey-Nagel, Düren, Germany) for RNA-seq and ISOGEN (Nippongene, Tokyo, Japan) for qPCR analyses.

For RNA-seq, homogenization was conducted for each tissue using an electric automatic homogenizer (T10 + S10N-5G, IKA Works, Staufen, Germany), and the samples were then treated with spin columns and DNase according to the manufacturer’s instructions. RNA samples (eight sets) from individuals of the S and L strains were prepared, and three sets were selected from among the high-quality RNAs via the following tests. The extracted RNA was qualitatively and quantitatively examined at 230, 260, and 280 nm by a spectrophotometer (Nanodrop^TM^ 2000, Thermo Fisher Scientific, Waltham, MA, USA) and RNA integrity was determined by using the Agilent RNA 6000 Nano Kit (Agilent Technologies) and an Agilent Bioanalyzer 2100 (Agilent Technologies, Santa Clara, CA, USA).

### cDNA library preparation and sequencing

Using the TruSeq RNA Library Preparation Kit v2 (Illumina, San Diego, CA, USA), library preparation was conducted from 500 ng of total RNA according to the manufacturer’s protocol. Super-Script III reverse transcriptase (Invitrogen, Carlsbad, CA, USA) was used to synthesize first-strand cDNAs with random hexamers and short fragmented mRNAs. Double-stranded cDNAs with P5 and P7 adaptor sequences at both ends were amplified by 15 cycles of high-fidelity PCR. After second-stranded cDNA synthesis, dA-tailed and end-repaired fragments were combined by sequencing adaptors. The adaptor-ligated fragments of cDNA were amplified by 15 cycles of PCR, and then AM-Pure XP magnetic beads (Beckman Coulter, Brea, CA, USA) were used to clean up the products. Library concentration and quality was assessed with the Agilent DNA 1000 Kit using an Agilent Bioanalyzer 2100 (Agilent Technologies). In addition, the library concentration was precisely determined using the KAPA Library Quantification Kit (Kapa Biosystems, Wilmington, MA, USA) and a Step-One-Plus real-time PCR-System (Applied Biosystems Laboratories, Foster City, CA, USA).

We sequenced the cDNA library with an Illumina HiSeq 2500 platform (Illumina) using two lanes of a flow cell (6 samples per lane) and producing 100-bp single reads. Reads were generated in FASTQ format using the conversion software bcl2fastq2 (Illumina, version 2.18). We submitted the read data to the Read Archive of DDBJ (accession number DRA007602).

### Analysis of differentially expressed genes

Using TagDust (version 1.13), P5 and P7 adaptors sequence containing raw reads were removed. In addition, we used FASTX-Toolkit, ver. 0.0.13.2, to clip and trim the first 13 bp of each read and to clip low-quality reads. We used the following quality-filtering parameters: (i) minimum quality score, 20, and (ii) minimum percentage of bases with [−q] quality, 80. Then, the clean-read data were mapped using Tophat2 (version 2.0.12) with Bowtie2 to the reference genome of *T*. *castaneum* (Tcas5.2) that was obtained from the NCBI genome database (https://www.ncbi.nlm/nih.gov/). The expression level of each transcript was calculated by normalizing as “fragments per kilobase of exon per million reads mapped (FPKM)” using Cufflinks (ver. 2.2.1). Statistical significance was calculated by comparison between the strains using the Cuffdiff module of Cufflinks. Finally, DEGs with significant thresholds were identified based on fold changes (FC ≥ 1.5 or ≤ 0.67), and the false discovery rate, i.e., FDR, was modified with a 0.05 percent level of the *P*-value (q < 0.05).

### Gene ontology and pathway enrichment analysis

Gene IDs for each transcript were associated with pathway analysis and the previously mentioned enrichment analysis. Some of the gene IDs not listed in the Tcas 5.2 reference genome were downloaded from the NCBI Refseq database (https://www.ncbi.nlm.nih.gov/gene/). GO and KEGG pathway enrichment analyses of the DEGs from the strains were conducted using the web-based tool DAVID 6.8 (https://david.ncifcrf.gov). The enriched GO and KEGG terms with the pathways were statistically analyzed using the modified Fisher’s exact test at a 0.05 percent level of probability (*P* < 0.05) as determined by the tool.

### Pathway analysis

KEGG metabolic maps (https://www.kegg.jp/kegg/kegg1.html) based on FCs between the L and S strains were highlighted using the R/Bioconductor package “Pathview” to compare expression levels during death feigning^21–23^.

### qPCR analyses

For qPCR analyses, each tissue was minced by sterilized scissors in ISOGEN solution. RNA extraction was performed according to the manufacturer’s instructions. The extracted RNA was treated with DNase (RT Grade for Heat Stop, Nippongene) at 37 °C for 15 min and then mixed with stop solution at 70 °C for 10 min. The extracted RNA was qualitatively and quantitatively examined by the same procedure used for RNA-seq. DNase-treated RNA (300 ng) was transcribed using the High-Capacity cDNA Reverse Transcription Kit (Applied Biosystems) for single-strand cDNA synthesis according to the manufacturer’s instructions.

Two genes associated with tyrosine metabolism [tyrosine aminotransferase (*Tat*): *Tctat* and 4-hydroxyphenyl-pyruvate dioxygenase (*Hpd*): *Tchpd*] and two genes associated with dopamine metabolism [DOPA decarboxylase (*Ddc*): *Tcddc* and *N*-acetyltransferase (*Nat*): *Tcnat*] were selected as target genes (S3 Table). Two reference genes [actin (*Act*): *Tcact* and tbp associated factor (*Tbpaf*): *Tctbpaf* were examined with the appropriate primer sets (S3 Table). The primer sequences for the target and reference genes were designed with Primer 3 Plus (http://www.bioinformatics.nl/cgi-bin/primer3plus/primer3plus.cgi/), but those for *Tcddc* were designed according to the method of Arakane *et al*.^23^. Standard curves were generated to examine the efficiency of the target and reference (dilutions of 1, 1/5, 1/10, 1/100). The template for generation of these curves was obtained from cDNAs of the S strain. The relative quantification of cDNAs was performed using the KAPA SYBR ® FAST qPCR Kit (KAPA Biosystems, Nippon Genetics, Tokyo, Japan) with a real-time PCR system (Eco, Illumina, San Diego, CA, USA). Each reaction mixture (total volume 20 μl) consisted of 10 μl of KAPA SYBR Fast qPCR mix, 0.4 μl of each of the forward and reverse primers (10 μM), 7.2 μl of RNase-free water, and 2 μl of the cDNA template. The temperature profile for amplification of the reference gene with target gene fragments was as follows: 95 °C for 1 min, followed by 40 cycles of 95 °C for 3 s and 60 °C for 20 s. Each sample was repeated twice in a single run. The amplification of single products was confirmed by dissociation curve analysis with the real-time PCR system.

We recorded the quantification cycle (Cq) number at which each reaction crossed a threshold fluorescent intensity within the linear portion of the amplification curve to estimate the levels of mRNA expression for each gene. The suitability of two reference genes as internal control genes (*Tcact* and *Tctbpaf*) was evaluated and examined using the t-test. The Cq values for *Tcact* were mostly stable and not significantly different among the developmental stages examined (t-test, *Tcact*: t = 0.253, *P* = 0.402, N_L_ = N_S_ = 10; *Tctbpaf*: t = −1.31, *P* = 0.103, N_L_ = N_S_ = 10). Therefore, we confirmed the normalized expression levels for the target genes with the expression levels of *Tcact*.

## Supplementary information

S1 Table

S2 Table

S3 Table

## Data Availability

The datasets generated during and/or analyzed during the current study are available from the corresponding author on reasonable request.
