# Supplementary material for: Transcriptomic comparison between beetle strains selected for short and long durations of death feigning
Source: Sci Rep. 2019 Sep 30;9:14001. doi: 10.1038/s41598-019-50440-5 (PMC6768993; doi:10.1038/s41598-019-50440-5)
Supplement: Supplementary file 1 — S1 Table [file 41598_2019_50440_MOESM1_ESM.pdf]

Title page for S1 Table

The title of manuscript: Transcriptomic comparison between beetle strains selected for short and long durations of death feigning.

The author list:
Hironobu Uchiyama
Ken Sasaki
Shogo Hinosawa
Keisuke Tanaka
Kentarou Matsumura
Shunsuke Yajima
Takahisa Miyatake

S1 Table. Sequence summary of each sample.

Sample ID	Description	Platform	Sequencing protocol	Total number of sequence	Sequence length	%GC
SAMD00151344	DF Long line 1	HiSeq2500	Single read 100 cycles	26,956,970	35-100	40
SAMD00151345	DF Long line 2	HiSeq2500	Single read 100 cycles	24,079,235	35-100	41
SAMD00151346	DF Long line 3	HiSeq2500	Single read 100 cycles	27,113,745	35-100	41
SAMD00151347	DF Short line1	HiSeq2500	Single read 100 cycles	27,657,283	35-100	43
SAMD00151348	DF Short line2	HiSeq2500	Single read 100 cycles	29,495,302	35-100	44
SAMD00151349	DF Short line3	HiSeq2500	Single read 100 cycles	23,963,878	35-100	44
