# Supplementary material for: Transcriptomic comparison between beetle strains selected for short and long durations of death feigning
Source: Sci Rep. 2019 Sep 30;9:14001. doi: 10.1038/s41598-019-50440-5 (PMC6768993; doi:10.1038/s41598-019-50440-5)
Supplement: Supplementary file 2 — S2 Table [file 41598_2019_50440_MOESM2_ESM.pdf]

Title page for S2 Table

The title of : Transcriptomic comparison between beetle strains selected formanuscript short and long durations of death feigning.

The author list: Hironobu Uchiyama, Ken Sasaki, Shogo Hinosawa, Keisuke Tanaka, Kentarou Matsumura, Shunsuke Yajima, Takahisa Miyatake

S2 Table. List of differentially expressed genes between the L and S strains.

| # | LOC name | Region | Gene ID | Description | FPKM for L | FPKM for S | log2(Fold change+1) | P-value | FDR |
|---|---|---|---|---|---|---|---|---|---|
| 1 | LOC107398286 | NC_007417.3:1379768-1380361 | 107398286 | uncharacterized LOC107398286 | 52.2821 | 0.502158 | 5.148542473 | 0.00105 | 0.0470692 |
| 2 | - | NW_015452025.1:2991-3878 | - | - | 25.5962 | 0.26362 | 4.39558555 | 0.0006 | 0.0297494 |
| 3 | P41 | NC_007418.3:10353706-10354992 | 661254 | serine protease P41 | 85.9659 | 1.24604 | 5.274994294 | 5.00E-05 | 0.00352415 |
| 4 | LOC657769 | NW_015451510.1:1505-3305 | 657769 | facilitated trehalose transporter Tret1 | 19.1283 | 0.688605 | 3.575321534 | 5.00E-05 | 0.00352415 |
| 5 | LOC660420 | NC_007423.3:9398892-9400590 | 660420 | equilibrative nucleoside transporter 3 | 23.1626 | 1.15738 | 3.485423484 | 5.00E-05 | 0.00352415 |
| 6 | LOC664395 | NC_007417.3:8685030-8689026 | 664395 | alpha-amylase-like | 6.53327 | 0.42944 | 2.397826173 | 5.00E-05 | 0.00352415 |
| 7 | LOC655017 | NW_015450971.1:3368-5214 | 655017 | moesin/ezrin/radixin homolog 1 | 7.75219 | 0.692362 | 2.370605861 | 5.00E-05 | 0.00352415 |
| 8 | LOC655071 | NC_007423.3:4594602-4595685 | 655071 | uncharacterized LOC655071 | 2.7097 | 0.244532 | 1.575699196 | 0.0009 | 0.0416491 |
| 9 | LOC103312472 | NC_007418.3:25460046-25495251 | 103312472 | uncharacterized LOC103312472 | 28.4757 | 2.9537 | 2.898250764 | 5.00E-05 | 0.00352415 |
| 10 | H115 | NC_007420.3:1471291-1478664 | 657133 | serine protease H115 | 11.7177 | 1.23055 | 2.511366389 | 0.00085 | 0.0395751 |
| 11 | - | NC_007423.3:54121-55236 | - | - | 12.8197 | 1.37677 | 2.539652092 | 5.00E-05 | 0.00352415 |
| 12 | LOC103314782 | NW_015450575.1:82793-86861 | 103314782 | uncharacterized | 109.221 | 13.0229 | 2.97454248 | 5.00E-05 | 0.00352415 |
| 13 | LOC657849 | NW_015451510.1:6335-7969 | 657849 | facilitated trehalose transporter Tret1 | 10.8051 | 1.29803 | 2.360940725 | 5.00E-05 | 0.00352415 |
| 14 | LOC100142014 | NW_015452111.1:26-1007 | 100142014 | uncharacterized LOC100142014 | 4.30895 | 0.552973 | 1.773393807 | 5.00E-05 | 0.00352415 |
| 15 | LOC103314746 | NC_007422.5:7413296-7414494 | 103314746 | uncharacterized LOC103314746 | 6.57533 | 0.84491 | 2.037758298 | 5.00E-05 | 0.00352415 |
| 16 | LOC103312957 | NC_007420.3:5088956-5091766 | 103312957 | adhesion G-protein coupled receptor G2 | 3.29397 | 0.450013 | 1.566246277 | 5.00E-05 | 0.00352415 |
| 17 | LOC103312900 | NC_007420.3:3289609-3290934 | 103312900 | uncharacterized LOC103312900 | 10.0159 | 1.39886 | 2.1991665 | 5.00E-05 | 0.00352415 |
| 18 | En | NC_007422.5:12546768-12551040 | 661645 | engrailed | 1.75671 | 0.246453 | 1.145119023 | 0.0001 | 0.00663971 |
| 19 | - | NC_007417.3:5261973-5262712 | - | - | 6.67451 | 0.964678 | 1.965781759 | 0.00015 | 0.00949185 |
| 20 | LOC103312546 | NC_007419.2:733210-738581 | 103312546 | uncharacterized LOC103312546 | 4.68397 | 0.709 | 1.733746543 | 0.00015 | 0.00949185 |
| 21 | p98; p97 | NC_007422.5:7350928-7355730 | 662682 | serine protease P98 | 110.405 | 16.9 | 2.637782492 | 5.00E-05 | 0.00352415 |
| 22 | LOC658555 | NC_007416.3:1023768-1025032 | 658555 | phospholipase A1 | 40.6537 | 6.27272 | 2.517877714 | 5.00E-05 | 0.00352415 |
| 23 | LOC103314809 | NC_007423.3:48118-53973 | 103314809 | hypothetical protein | 0.901306 | 0.148064 | 0.727787672 | 5.00E-05 | 0.00352415 |
| 24 | P162 | NC_007421.3:3491859-3492749 | 658877 | serine protease P162 | 324.702 | 54.0901 | 2.563687592 | 5.00E-05 | 0.00352415 |
| 25 | LOC657602 | NC_007418.3:11094440-11095409 | 657602 | glutathione S-transferase | 92.1045 | 15.5342 | 2.493397656 | 5.00E-05 | 0.00352415 |
| 26 | P167 | NW_015451646.1:123355-125194 | 655811 | brachyurin | 133.333 | 22.6791 | 2.504127605 | 5.00E-05 | 0.00352415 |
| 27 | LOC658800 | NC_007421.3:215514-217461 | 658800 | fatty acyl-CoA reductase 1 | 3.83549 | 0.721094 | 1.490336197 | 5.00E-05 | 0.00352415 |
| 28 | - | NC_007420.3:3291809-3294329 | - | - | 8.07394 | 1.54969 | 1.83140727 | 0.00075 | 0.0357178 |
| 29 | LOC103313637 | NC_007422.5:8621702-8632850 | 103313637 | uncharacterized LOC103313637 | 3.94685 | 0.779097 | 1.475364983 | 0.0002 | 0.0120247 |
| 30 | LOC103312194 | NC_007417.3:14780923-14782632 | 103312194 | uncharacterized LOC103312194 | 279.889 | 56.7282 | 2.282651981 | 5.00E-05 | 0.00352415 |
| 31 | LOC103314784 | NC_007417.3:13814650-13958477 | 103314784 | choline transporter-like protein 1 | 15.1393 | 3.08012 | 1.983894518 | 0.00105 | 0.0470692 |
| 32 | - | NC_007422.5:15644810-15650547 | - | - | 1.55942 | 0.336451 | 0.937409969 | 5.00E-05 | 0.00352415 |
| 33 | H106 | NC_007420.3:1492710-1493841 | 657444 | serine protease H106 | 36.19 | 7.84701 | 2.071652889 | 5.00E-05 | 0.00352415 |
| 34 | LOC655001 | NC_007422.5:1534305-1564073 | 655001 | cytochrome P450 4C1 | 325.439 | 71.757 | 2.165655465 | 5.00E-05 | 0.00352415 |
| 35 | LOC103314085 | NC_007423.3:12238186-12238830 | 103314085 | uncharacterized LOC103314085 | 41.5406 | 9.34823 | 2.039456357 | 5.00E-05 | 0.00352415 |
| 36 | - | NC_007418.3:16224511-16225266 | - | - | 5.56382 | 1.25218 | 1.54321354 | 0.0003 | 0.0169681 |
| 37 | LOC103314667 | NW_015451779.1:18-981 | 103314667 | RNA-directed DNA polymerase from mobile element jockey-like | 75.816 | 17.1427 | 2.082017667 | 5.00E-05 | 0.00352415 |
| 38 | - | NW_015450592.1:2313-2963 | - | - | 18.8732 | 4.35037 | 1.893113627 | 5.00E-05 | 0.00352415 |

| | | | | | | | | | |
|---|---|---|---|---|---|---|---|---|---|
| 39 | - | NW_015451028.1:939-1324 | - | - | 52.9167 | 12.2117 | 2.028916083 | 5.00E-05 | 0.00352415 |
| 40 | LOC661967 | NC_007419.2:10352167-10455479 | 661967 | UDP-glucuronosyltransferase 2B20 | 177.795 | 42.1197 | 2.051887346 | 5.00E-05 | 0.00352415 |
| 41 | LOC100142031 | NC_007423.3:6587154-6588963 | 100142031 | sulfhydryl oxidase 2 | 8.20063 | 1.98056 | 1.626149235 | 5.00E-05 | 0.00352415 |
| 42 | LOC659565 | NC_007425.3:6902757-6908923 | 659565 | cathepsin L precursor | 1.93697 | 0.475683 | 0.992945688 | 0.00065 | 0.0319518 |
| 43 | LOC660784 | NC_007422.5:4982936-4984551 | 660784 | translation initiation factor IF-2 | 4.01513 | 0.999779 | 1.326446525 | 0.00025 | 0.014684 |
| 44 | LOC107397485 | NC_007418.3:2823010-2827641 | 107397485 | uncharacterized LOC107397485 | 2.77274 | 0.714714 | 1.137644713 | 0.00085 | 0.0395751 |
| 45 | LOC664406 | NC_007419.2:5876706-5879343 | 664406 | double-stranded RNA-binding protein Staufen homolog 2 | 13.9026 | 3.62987 | 1.686520464 | 5.00E-05 | 0.00352415 |
| 46 | LOC107399160 | NW_015451916.1:2097-6354 | 107399160 | uncharacterized LOC107399160 | 12.0641 | 3.16001 | 1.650948838 | 5.00E-05 | 0.00352415 |
| 47 | LOC107397936 | NC_007420.3:1468245-1469916 | 107397936 | brachyurin-like | 9.28293 | 2.43156 | 1.583314917 | 5.00E-05 | 0.00352415 |
| 48 | LOC658629 | NW_015451776.1:33994-49469 | 658629 | excitatory amino acid transporter | 38.19 | 10.2554 | 1.799868244 | 5.00E-05 | 0.00352415 |
| 49 | LOC103314558 | NW_015451937.1:352665-366702 | 103314558 | uncharacterized LOC103314558 | 4.35173 | 1.1733 | 1.300117996 | 0.0001 | 0.00663971 |
| 50 | - | NC_007420.3:12858-13232 | - | - | 16.973 | 4.59116 | 1.684611704 | 0.0002 | 0.0120247 |
| 51 | LOC661353 | NC_007416.3:4610333-4618961 | 661353 | IQ motif and SEC7 domain-containing protein 1 | 9.46672 | 2.56825 | 1.552520807 | 0.00065 | 0.0319518 |
| 52 | LOC103313572 | NC_007422.5:6600363-6601159 | 103313572 | uncharacterized LOC103313572 | 5.6366 | 1.53145 | 1.390480334 | 0.0004 | 0.0218162 |
| 53 | LOC664571 | NC_007422.5:9814138-9816152 | 664571 | beta-hexosaminidase subunit alpha | 28.4495 | 7.88636 | 1.728578646 | 5.00E-05 | 0.00352415 |
| 54 | LOC103314151 | NC_007418.3:31269917-31273035 | 103314151 | caspase-1-like | 124.418 | 35.081 | 1.79743319 | 5.00E-05 | 0.00352415 |
| 55 | CYP6BK14 | NC_007421.3:2191548-2200968 | 658048 | cytochrome P450 6a9 | 30.1745 | 8.58782 | 1.701091692 | 5.00E-05 | 0.00352415 |
| 56 | LOC107398944 | NW_015450750.1:304-5356 | 107398944 | uncharacterized LOC107398944 | 13.3049 | 3.79053 | 1.57825223 | 5.00E-05 | 0.00352415 |
| 57 | LOC100141724 | NC_007420.3:9455633-9460403 | 100141724 | uncharacterized LOC100141724 | 13.3521 | 3.82245 | 1.573423662 | 5.00E-05 | 0.00352415 |
| 58 | Cht11 | NC_007422.5:3380907-3384466 | 661649 | chitinase 11 | 160.253 | 46.7731 | 1.7550556 | 5.00E-05 | 0.00352415 |
| 59 | LOC662574 | NC_007420.3:4834999-4845524 | 662574 | multidrug resistance-associated protein 4 | 16.9259 | 4.99384 | 1.580493076 | 5.00E-05 | 0.00352415 |
| 60 | LOC654890 | NC_007424.3:9-2543 | 654890 | condensin complex subunit 2 | 1.89934 | 0.561078 | 0.893181901 | 0.00015 | 0.00949185 |
| 61 | LOC103314327 | NC_007424.3:13863233-13864178 | 103314327 | uncharacterized LOC103314327 | 33.4387 | 9.8932 | 1.66060286 | 0.0001 | 0.00663971 |
| 62 | LOC657270 | NC_007421.3:3334661-3340656 | 657270 | farnesol dehydrogenase | 6.1236 | 1.86657 | 1.313281 | 5.00E-05 | 0.00352415 |
| 63 | LOC107399097 | NW_015451667.1:314-924 | 107399097 | uncharacterized LOC107399097 | 32.5255 | 10.2772 | 1.571849941 | 0.0001 | 0.00663971 |
| 64 | Ctlp-6a | NC_007423.3:9384852-9386634 | 660544 | chymotrypsin-like proteinase 6A precursor | 580.719 | 183.663 | 1.655427596 | 5.00E-05 | 0.00352415 |
| 65 | H112 | NC_007420.3:1445095-1446291 | 656795 | serine protease H112 | 711.659 | 227.474 | 1.641181929 | 5.00E-05 | 0.00352415 |
| 66 | LOC656564 | NW_015450233.1:639257-647866 | 656564 | alanine--glyoxylate aminotransferase 2-like | 305.798 | 98.3532 | 1.626650738 | 5.00E-05 | 0.00352415 |
| 67 | LOC100142621 | NC_007424.3:5810259-5814549 | 100142621 | uncharacterized LOC100142621 | 5.36544 | 1.72632 | 1.223305334 | 5.00E-05 | 0.00352415 |
| 68 | LOC662738 | NC_007422.5:491494-493570 | 662738 | tenecin-3 | 3764.71 | 1211.94 | 1.634413714 | 5.00E-05 | 0.00352415 |
| 69 | LOC103313776 | NC_007423.3:2629468-2630331 | 103313776 | 39S ribosomal protein L34, mitochondrial | 97.6414 | 31.5248 | 1.60065309 | 5.00E-05 | 0.00352415 |
| 70 | LOC107397745 | NC_007419.2:5039546-5040153 | 107397745 | putative glycine-rich cell wall structural protein 1 | 8.79459 | 2.86334 | 1.342136453 | 0.0006 | 0.0297494 |
| 71 | H110 | NC_007420.3:1394616-1395757 | 656549 | serine protease H110 | 71.2404 | 23.2313 | 1.575934074 | 5.00E-05 | 0.00352415 |
| 72 | LOC655516 | NC_007418.3:26705775-26718077 | 655516 | CYCLE | 4.22001 | 1.38464 | 1.130281086 | 5.00E-05 | 0.00352415 |
| 73 | LOC103312887 | NC_007420.3:28067-61231 | 103312887 | uncharacterized LOC103312887 | 3.13478 | 1.03691 | 1.021428333 | 5.00E-05 | 0.00352415 |
| 74 | Pmp5-a | NC_007418.3:628675-630154 | 662504 | peritrophic matrix protein 5-A | 1511.45 | 508.411 | 1.569985431 | 5.00E-05 | 0.00352415 |
| 75 | LOC100141997 | NC_007421.3:3075173-3079776 | 100141997 | uncharacterized LOC100141997 | 19.0399 | 6.49399 | 1.419069352 | 5.00E-05 | 0.00352415 |
| 76 | LOC103312772 | NW_015451146.1:6-6203 | 103312772 | uncharacterized LOC103312772 | 3.53287 | 1.22614 | 1.025880461 | 5.00E-05 | 0.00352415 |
| 77 | LOC663233 | NC_007422.5:13636232-13653424 | 663233 | beta-mannosidase-like | 50.9604 | 18.1518 | 1.439932545 | 5.00E-05 | 0.00352415 |
| 78 | LOC662265 | NC_007422.5:13799677-13816250 | 662265 | 40S ribosomal protein S20 | 3012.95 | 1073.82 | 1.487560411 | 5.00E-05 | 0.00352415 |
| 79 | LOC657457 | NW_015450689.1:72670-73558 | 657457 | pathogenesis-related protein 5 | 967.362 | 355.99 | 1.439662805 | 5.00E-05 | 0.00352415 |
| 80 | LOC662599 | NC_007417.3:12004643-12007708 | 662599 | protein Malvolio | 25.8335 | 9.62938 | 1.335977795 | 5.00E-05 | 0.00352415 |
| 81 | OBP02; TcOBP6E | NC_007421.3:4270904-4271453 | 103313402 | uncharacterized LOC103313402 | 103.94 | 38.8519 | 1.396844281 | 5.00E-05 | 0.00352415 |
| 82 | LOC661631 | NC_007419.2:7874608-7876123 | 661631 | lipase 1 | 272.785 | 102.308 | 1.406091429 | 5.00E-05 | 0.00352415 |

| | | | | | | | | | |
|---|---|---|---|---|---|---|---|---|---|
| 83 | LOC663761 | NC_007417.3:7304084-7351652 | 663761 | neurotrimin | 35.0154 | 13.1762 | 1.345143065 | 5.00E-05 | 0.00352415 |
| 84 | LOC663779 | NC_007422.5:11443752-11445913 | 663779 | armadillo repeat-containing protein 6 homolog | 16.9436 | 6.38819 | 1.280176492 | 5.00E-05 | 0.00352415 |
| 85 | LOC103312285 | NC_007418.3:2814876-2817198 | 103312285 | hypothetical protein | 21.085 | 7.98635 | 1.297259674 | 0.0003 | 0.0169681 |
| 86 | LOC660599 | NC_007420.3:9972389-9981631 | 660599 | multidrug resistance-associated protein 4 | 10.7592 | 4.10827 | 1.202883228 | 0.00055 | 0.0281853 |
| 87 | LOC103313234 | NC_007420.3:14373667-14374332 | 103313234 | uncharacterized LOC103313234 | 12.3884 | 4.79116 | 1.209059298 | 0.0008 | 0.037707 |
| 88 | Lyz | NC_007418.3:24192177-24210829 | 658610 | lysozyme | 366.166 | 143.561 | 1.344754079 | 5.00E-05 | 0.00352415 |
| 89 | LOC107398028 | NC_007420.3:13176967-13177792 | 107398028 | odorant binding protein 26 | 77.534 | 30.5099 | 1.317512204 | 5.00E-05 | 0.00352415 |
| 90 | LOC655392 | NC_007418.3:11409032-11418268 | 655392 | protein henna | 80.6237 | 31.9052 | 1.310672519 | 5.00E-05 | 0.00352415 |
| 91 | LOC656754 | NC_007418.3:27903750-28035531 | 656754 | lysosomal alpha-mannosidase | 122.125 | 48.332 | 1.319528042 | 0.0002 | 0.0120247 |
| 92 | LOC655801 | NC_007424.3:1353531-1382237 | 655801 | high affinity cationic amino acid transporter 1 | 17.9432 | 7.1147 | 1.223070396 | 0.0002 | 0.0120247 |
| 93 | LOC659891 | NC_007422.5:3955419-3957350 | 659891 | forkhead box protein D3 | 10.3091 | 4.10524 | 1.147433432 | 5.00E-05 | 0.00352415 |
| 94 | - | NC_007418.3:16223589-16224312 | - | - | 9.77493 | 3.92195 | 1.13037659 | 0.00035 | 0.0194127 |
| 95 | - | NW_015452251.1:11-762 | - | - | 81.3587 | 32.9418 | 1.278857982 | 5.00E-05 | 0.00352415 |
| 96 | LOC103313070 | NC_007420.3:9963200-9966670 | 103313070 | multidrug resistance-associated protein 4 | 6.60952 | 2.67779 | 1.048966348 | 0.0003 | 0.0169681 |
| 97 | LOC103313186 | NC_007420.3:11563173-11563908 | 103313186 | hypothetical protein | 34.7562 | 14.0855 | 1.245030907 | 5.00E-05 | 0.00352415 |
| 98 | Gpa2 | NC_007424.3:11271960-11272443 | 659603 | glycoprotein hormone alpha 2 | 16.3984 | 6.70151 | 1.175741397 | 0.0011 | 0.0486442 |
| 99 | LOC661035 | NC_007417.3:11485320-11487090 | 661035 | sorbitol dehydrogenase | 51.8167 | 21.1846 | 1.251435624 | 5.00E-05 | 0.00352415 |
| 100 | LOC662228 | NC_007422.5:12036815-12038954 | 662228 | aromatic amino acid aminotransferase DDB_G0287711 | 125.95 | 52.1341 | 1.25655045 | 5.00E-05 | 0.00352415 |
| 101 | LOC655628 | NC_007424.3:14524749-14529705 | 655628 | retinol dehydrogenase 11 | 99.6015 | 41.2924 | 1.250181479 | 5.00E-05 | 0.00352415 |
| 102 | LOC661309 | NC_007425.3:4272561-4275637 | 661309 | double-stranded RNA-specific editase Adar | 14.3085 | 5.9376 | 1.141824361 | 5.00E-05 | 0.00352415 |
| 103 | LOC103313095 | NC_007420.3:8434484-8435463 | 103313095 | hypothetical protein | 697.355 | 290.265 | 1.261628249 | 5.00E-05 | 0.00352415 |
| 104 | LOC655554 | NC_007423.3:630271-638678 | 655554 | Niemann-Pick C1 protein | 56.7915 | 23.7143 | 1.225511271 | 5.00E-05 | 0.00352415 |
| 105 | LOC661924 | NC_007420.3:4623499-4626668 | 661924 | rhodopsin 1/6-like | 1611.69 | 674.602 | 1.255223638 | 5.00E-05 | 0.00352415 |
| 106 | LOC662513 | NC_007420.3:13424740-13425890 | 662513 | endocuticle structural glycoprotein SgAbd-8 | 95.3982 | 40.6846 | 1.209491717 | 5.00E-05 | 0.00352415 |
| 107 | LOC656188 | NC_007420.3:14740830-14742118 | 656188 | regucalcin | 46.2716 | 19.7615 | 1.187063015 | 5.00E-05 | 0.00352415 |
| 108 | Cpap3-d2 | NC_007417.3:14931519-14937394 | 662181 | cuticular protein analogous to peritrophins 3-D2 | 25.2063 | 10.9014 | 1.138782385 | 5.00E-05 | 0.00352415 |
| 109 | LOC103313294 | NC_007418.3:19307486-19310005 | 103313294 | uncharacterized LOC103313294 | 12.443 | 5.38244 | 1.074675157 | 5.00E-05 | 0.00352415 |
| 110 | LOC661007 | NC_007423.3:13829831-13837015 | 661007 | sodium-independent sulfate anion transporter | 21.4903 | 9.34143 | 1.120867212 | 5.00E-05 | 0.00352415 |
| 111 | LOC655199 | NC_007420.3:3549348-3550655 | 655199 | putative thiosulfate sulfurtransferase, mitochondrial | 16.0454 | 6.98422 | 1.094159076 | 5.00E-05 | 0.00352415 |
| 112 | CYP301B1 | NC_007419.2:8147128-8149464 | 662872 | cytochrome P450 301B1 | 6.34127 | 2.78836 | 0.954456229 | 0.00035 | 0.0194127 |
| 113 | LOC100142166 | NC_007424.3:15004201-15015166 | 100142166 | sortilin-related receptor | 9.50901 | 4.18693 | 1.018673962 | 5.00E-05 | 0.00352415 |
| 114 | Cda2 | NC_007420.3:5690326-5696317 | 657879 | chitin deacetylase 2 | 16.8791 | 7.48447 | 1.075377669 | 5.00E-05 | 0.00352415 |
| 115 | - | NW_015451937.1:344610-351959 | - | - | 9.82392 | 4.37748 | 1.009220922 | 5.00E-05 | 0.00352415 |
| 116 | LOC662282 | NC_007416.3:5004669-5011572 | 662282 | protein dispatched | 10.8431 | 4.84512 | 1.018742216 | 0.0004 | 0.0218162 |
| 117 | - | NC_007425.3:5090887-5094023 | - | - | 274.109 | 124.066 | 1.1373137 | 0.0003 | 0.0169681 |
| 118 | LOC655432 | NC_007418.3:26698761-26702399 | 655432 | purine nucleoside phosphorylase | 145.397 | 65.8867 | 1.130094716 | 5.00E-05 | 0.00352415 |
| 119 | LOC103314416 | NC_007425.3:4177500-4180560 | 103314416 | major facilitator superfamily domain-containing protein 9 | 29.5831 | 13.4365 | 1.083013634 | 0.0005 | 0.0259129 |
| 120 | LOC656943 | NC_007421.3:100388-102720 | 656943 | putative fatty acyl-CoA reductase CG5065 | 26.1632 | 11.921 | 1.071935719 | 0.00035 | 0.0194127 |
| 121 | LOC660051 | NC_007421.3:9329348-9338327 | 660051 | facilitated trehalose transporter Tret1 | 62.3055 | 28.6112 | 1.09618789 | 0.0001 | 0.00663971 |
| 122 | LOC662799 | NC_007420.3:13454931-13455834 | 662799 | cuticular protein | 91.3492 | 41.9589 | 1.104142411 | 5.00E-05 | 0.00352415 |
| 123 | - | NC_007422.5:3374943-3376651 | - | - | 38.266 | 17.5895 | 1.078792672 | 5.00E-05 | 0.00352415 |
| 124 | LOC655457 | NC_007424.3:19100-23233 | 655457 | facilitated trehalose transporter Tret1 | 26.5642 | 12.3113 | 1.050144257 | 5.00E-05 | 0.00352415 |
| 125 | LOC100124592 | NC_007418.3:10621195-10631319 | 100124592 | aspartate 1-decarboxylase | 11.4935 | 5.3873 | 0.96789958 | 5.00E-05 | 0.00352415 |
| 126 | LOC103313511 | NC_007422.5:497209-497796 | 103313511 | ctenidin-1 | 34.4555 | 16.3957 | 1.027278708 | 0.00045 | 0.0239168 |

| | | | | | | | | | |
|---|---|---|---|---|---|---|---|---|---|
| 127 | LOC661923 | NC_007419.2:7888979-7890342 | 661923 | lipase 3-like | 188.429 | 90.0108 | 1.057547551 | 5.00E-05 | 0.00352415 |
| 128 | LOC657931 | NW_015451510.1:9032-14230 | 657931 | facilitated trehalose transporter Tret1-2 homolog | 119.599 | 57.1615 | 1.052081562 | 5.00E-05 | 0.00352415 |
| 129 | LOC658392 | NC_007418.3:19715813-19736503 | 658392 | proton-coupled amino acid transporter 1 | 73.4753 | 35.3597 | 1.034421734 | 5.00E-05 | 0.00352415 |
| 130 | LOC661878 | NC_007419.2:7884209-7887092 | 661878 | lipase 3-like | 354.717 | 171.129 | 1.047239743 | 5.00E-05 | 0.00352415 |
| 131 | LOC661764 | NC_007422.5:5957498-5967074 | 661764 | ninjurin-2 | 120.025 | 58.0114 | 1.036239504 | 5.00E-05 | 0.00352415 |
| 132 | P96 | NC_007422.5:3177207-3178319 | 658137 | brachyurin | 265.291 | 128.431 | 1.040820471 | 5.00E-05 | 0.00352415 |
| 133 | - | NW_015451163.1:58-971 | - | - | 11.1149 | 5.39036 | 0.922813384 | 0.00085 | 0.0395751 |
| 134 | UV-opsin | NC_007418.3:10911917-10954172 | 658900 | opsin, ultraviolet-sensitive | 151.282 | 73.4773 | 1.031872745 | 5.00E-05 | 0.00352415 |
| 135 | LOC664194 | NC_007418.3:6596632-6599094 | 664194 | probable ATP-dependent RNA helicase DDX52 | 8.08049 | 3.92701 | 0.88205775 | 0.0005 | 0.0259129 |
| 136 | LOC662658 | NC_007424.3:13443648-13448593 | 662658 | 4-hydroxyphenylpyruvate dioxygenase | 517.829 | 254.711 | 1.020744899 | 5.00E-05 | 0.00352415 |
| 137 | LOC656983 | NC_007422.5:6833385-6834282 | 656983 | hypothetical protein | 691.433 | 340.564 | 1.019518556 | 5.00E-05 | 0.00352415 |
| 138 | LOC659580 | NC_007419.2:10032071-10033682 | 659580 | venom acid phosphatase Acph-1 | 304.625 | 150.18 | 1.015495266 | 5.00E-05 | 0.00352415 |
| 139 | LOC103313593 | NC_007422.5:3615164-3616462 | 103313593 | uncharacterized LOC103313593 | 79.2358 | 39.2097 | 0.996702517 | 5.00E-05 | 0.00352415 |
| 140 | Cyp4g14 | NC_007419.2:4413404-4415340 | 662217 | cytochrome P450 monooxygenase | 234.193 | 116.392 | 1.002511027 | 5.00E-05 | 0.00352415 |
| 141 | LOC663325 | NC_007417.3:9989620-9991074 | 663325 | probable phosphoserine aminotransferase | 152.021 | 75.7827 | 0.994876459 | 5.00E-05 | 0.00352415 |
| 142 | LOC103314523 | NC_007416.3:7718927-7720296 | 103314523 | uncharacterized LOC103314523 | 23.0959 | 11.5492 | 0.941192291 | 5.00E-05 | 0.00352415 |
| 143 | HEX1A | NC_007423.3:13938454-13940965 | 660335 | arylphorin | 5.66952 | 2.83945 | 0.796683275 | 0.00045 | 0.0239168 |
| 144 | LOC659104 | NC_007422.5:1194959-1197544 | 659104 | vanin-like protein 2 | 149.815 | 75.4235 | 0.98069169 | 5.00E-05 | 0.00352415 |
| 145 | LOC657162 | NC_007417.3:4984251-4985436 | 657162 | 1,5-anhydro-D-fructose reductase | 47.0419 | 23.8245 | 0.952528549 | 5.00E-05 | 0.00352415 |
| 146 | LOC103312668 | NC_007419.2:4994664-4995835 | 103312668 | hypothetical protein | 15.5251 | 7.86958 | 0.897721307 | 0.00045 | 0.0239168 |
| 147 | LOC654861 | NC_007418.3:20658743-20660124 | 654861 | iodotyrosine deiodinase 1 | 41.2268 | 20.9708 | 0.94257152 | 5.00E-05 | 0.00352415 |
| 148 | Ddc | NC_007420.3:9022378-9028416 | 662962 | dopa decarboxylase | 16.3908 | 8.35231 | 0.894929643 | 5.00E-05 | 0.00352415 |
| 149 | LOC107398182 | NC_007422.5:3052401-3054328 | 107398182 | eukaryotic translation initiation factor 5B-like | 10.7208 | 5.46701 | 0.857900298 | 0.0011 | 0.0486442 |
| 150 | Chp | NC_007417.3:8576783-8595515 | 652931 | cell surface protein chaoptin | 28.493 | 14.546 | 0.923829158 | 5.00E-05 | 0.00352415 |
| 151 | LOC659062 | NC_007419.2:4066202-4071560 | 659062 | 1-phosphatidylinositol 4,5-bisphosphate phosphodiesterase | 33.3892 | 17.0565 | 0.929437281 | 0.0003 | 0.0169681 |
| 152 | LOC661515 | NC_007422.5:8816451-8819563 | 661515 | elongation factor Tu | 143.833 | 74.13 | 0.946929349 | 0.0003 | 0.0169681 |
| 153 | LOC103314151 | NC_007418.3:31269917-31273035 | 103314151 | caspase-1-like | 59.0496 | 30.599 | 0.926275733 | 0.0007 | 0.0338289 |
| 154 | ImpL2 | NC_007421.3:8945769-8951756 | 657195 | ecdysone-inducible gene L2 | 79.4786 | 41.3323 | 0.926846333 | 5.00E-05 | 0.00352415 |
| 155 | LOC107398650 | NC_007424.3:9686219-9686420 | 107398650 | uncharacterized LOC107398650 | 2391.94 | 1250.67 | 0.934929974 | 5.00E-05 | 0.00352415 |
| 156 | LOC659093 | NC_007420.3:11252586-11253090 | 659093 | endocuticle structural glycoprotein SgAbd-5 | 155.783 | 81.8622 | 0.919983107 | 5.00E-05 | 0.00352415 |
| 157 | LOC660041 | NC_007420.3:6044023-6045427 | 660041 | Krueppel-like factor 11 | 94.9095 | 49.9855 | 0.911586713 | 5.00E-05 | 0.00352415 |
| 158 | LOC658874 | NC_007421.3:219042-220468 | 658874 | alpha-tocopherol transfer protein-like | 30.6796 | 16.3686 | 0.867072649 | 5.00E-05 | 0.00352415 |
| 159 | LOC662648 | NC_007422.5:3574137-3575741 | 662648 | spermine oxidase | 24.9704 | 13.3882 | 0.851982116 | 5.00E-05 | 0.00352415 |
| 160 | LOC660047 | NC_007421.3:2374864-2375715 | 660047 | uncharacterized LOC660047 | 30.6459 | 16.4969 | 0.854919262 | 0.0006 | 0.0297494 |
| 161 | Nag2 | NC_007418.3:3684052-3691642 | 658249 | beta-N-acetylglucosaminidase NAG2 | 32.9775 | 17.8294 | 0.851592677 | 0.0003 | 0.0169681 |
| 162 | LOC662670 | NC_007419.2:8123700-8129243 | 662670 | aldose reductase | 111.568 | 60.7089 | 0.867246284 | 0.00015 | 0.00949185 |
| 163 | Pmp1-b | NC_007421.3:3525434-3526482 | 659324 | peritrophic matrix protein 1-B | 825.445 | 452.515 | 0.865768543 | 5.00E-05 | 0.00352415 |
| 164 | LOC656561 | NC_007422.5:4149292-4151763 | 656561 | mannose-1-phosphate guanyltransferase alpha-A | 62.6382 | 34.3436 | 0.848444034 | 5.00E-05 | 0.00352415 |
| 165 | numb | NC_007424.3:6645097-6665968 | 661741 | protein numb | 24.382 | 13.3787 | 0.819872507 | 0.00105 | 0.0470692 |
| 166 | LOC657451 | NC_007425.3:5941152-5943554 | 657451 | UDP-glucuronosyltransferase 2B9 | 8.19401 | 4.50222 | 0.74068041 | 0.00065 | 0.0319518 |
| 167 | LOC661519 | NC_007424.3:3821084-3827221 | 661519 | facilitated trehalose transporter Tret1 | 45.4659 | 25.0317 | 0.835902822 | 0.00035 | 0.0194127 |
| 168 | H111 | NC_007420.3:1439218-1441500 | 656711 | serine protease H111 | 40.6491 | 22.5145 | 0.824734665 | 5.00E-05 | 0.00352415 |
| 169 | LOC103312286 | NC_007418.3:2817643-2821512 | 103312286 | lipopolysaccharide-induced tumor necrosis factor-alpha factor homolog | 168.014 | 93.2398 | 0.842734372 | 5.00E-05 | 0.00352415 |
| 170 | LOC659054 | NC_007420.3:1061308-1067260 | 659054 | cytochrome P450 345B1 | 38.1201 | 21.2545 | 0.813812971 | 0.00095 | 0.0436105 |

| | | | | | | | | | |
|---|---|---|---|---|---|---|---|---|---|
| 171 | LOC662035 | NC_007417.3:6104312-6109694 | 662035 | putative DNA helicase INO80 | 301.729 | 168.42 | 0.837422691 | 0.00015 | 0.00949185 |
| 172 | LOC662543 | NC_007420.3:6797945-6806048 | 662543 | inter-alpha-trypsin inhibitor heavy chain H4 | 163.378 | 91.2198 | 0.833868783 | 0.0004 | 0.0218162 |
| 173 | LOC658613 | NC_007418.3:15582811-15592736 | 658613 | glycine dehydrogenase (decarboxylating), mitochondrial | 16.6143 | 9.27909 | 0.777034593 | 5.00E-05 | 0.00352415 |
| 174 | - | NC_007418.3:15801610-15803677 | - | - | 30.29 | 16.997 | 0.797945221 | 5.00E-05 | 0.00352415 |
| 175 | LOC656432 | NC_007420.3:9468571-9470736 | 656432 | beta-galactosidase-1-like protein 2 | 117.96 | 66.2797 | 0.822233375 | 5.00E-05 | 0.00352415 |
| 176 | Pmp2-b | NC_007418.3:635588-636266 | 662632 | peritrophic matrix protein 2-B | 1670.46 | 941.89 | 0.825947453 | 0.0002 | 0.0120247 |
| 177 | LOC100141952 | NC_007421.3:5176885-5186355 | 100141952 | uncharacterized LOC100141952 | 47.4934 | 26.836 | 0.800836496 | 0.00085 | 0.0395751 |
| 178 | LOC664014 | NC_007419.2:3203324-3204205 | 664014 | uncharacterized LOC664014 | 269.934 | 153.219 | 0.812960933 | 5.00E-05 | 0.00352415 |
| 179 | LOC662129 | NC_007422.5:3466594-3468311 | 662129 | alpha-N-acetylgalactosaminidase | 68.5845 | 39.3028 | 0.787885908 | 5.00E-05 | 0.00352415 |
| 180 | LOC655252 | NW_015450233.1:23600-25618 | 655252 | kynurenine--oxoglutarate transaminase 3 | 38.1793 | 21.9066 | 0.774328285 | 5.00E-05 | 0.00352415 |
| 181 | LOC663674 | NC_007418.3:1075965-1077185 | 663674 | uncharacterized LOC663674 | 32.3425 | 18.5721 | 0.768563722 | 0.0001 | 0.00663971 |
| 182 | Th | NC_007418.3:26517372-26538072 | 654918 | tyrosine hydroxylase | 50.4661 | 29.0366 | 0.776900946 | 5.00E-05 | 0.00352415 |
| 183 | LOC103314048 | NC_007423.3:10035093-10036045 | 103314048 | uncharacterized LOC103314048 | 94.2427 | 54.3629 | 0.782689004 | 0.0007 | 0.0338289 |
| 184 | LOC655368 | NC_007423.3:9698633-9699893 | 655368 | uncharacterized LOC655368 | 80.429 | 46.8678 | 0.766487184 | 5.00E-05 | 0.00352415 |
| 185 | Csp12 | NC_007422.5:13945713-13946306 | 661270 | chemosensory protein 12 | 1471.04 | 859.881 | 0.773931142 | 5.00E-05 | 0.00352415 |
| 186 | LOC663235 | NC_007422.5:3641055-3642362 | 663235 | hypothetical protein | 93.6706 | 54.8118 | 0.76234629 | 5.00E-05 | 0.00352415 |
| 187 | LOC656567 | NC_007420.3:847715-857421 | 656567 | glutamate--cysteine ligase catalytic subunit | 42.3195 | 25.0197 | 0.735412263 | 5.00E-05 | 0.00352415 |
| 188 | LOC103313530 | NC_007422.5:179815-186554 | 103313530 | uncharacterized LOC103313530 | 64.7618 | 38.423 | 0.738212222 | 0.00035 | 0.0194127 |
| 189 | - | NC_007424.3:4463016-4463934 | - | - | 93.5573 | 55.5374 | 0.741983302 | 5.00E-05 | 0.00352415 |
| 190 | LOC657720 | NC_007419.2:3850272-3854338 | 657720 | open rectifier potassium channel protein 1 | 19.0301 | 11.3343 | 0.699493782 | 5.00E-05 | 0.00352415 |
| 191 | LOC100142298 | NC_007419.2:4301445-4302896 | 100142298 | uncharacterized LOC100142298 | 47.5589 | 28.4957 | 0.719231093 | 0.0002 | 0.0120247 |
| 192 | LOC103313498 | NC_007422.5:1065067-1067009 | 103313498 | uncharacterized LOC103313498 | 202.179 | 122.301 | 0.720566797 | 0.0001 | 0.00663971 |
| 193 | LOC661415 | NC_007419.2:63447-66167 | 661415 | nose resistant to fluoxetine protein 6-like | 39.6411 | 24.1044 | 0.694999206 | 0.0001 | 0.00663971 |
| 194 | LOC657080 | NW_015450233.1:406664-408759 | 657080 | venom carboxylesterase-6 | 93.183 | 57.0598 | 0.697927075 | 5.00E-05 | 0.00352415 |
| 195 | LOC664567 | NC_007422.5:9821196-9824417 | 664567 | beta-hexosaminidase subunit alpha | 104.754 | 64.2143 | 0.697451978 | 0.00015 | 0.00949185 |
| 196 | - | NW_015450951.1:3564-4038 | - | - | 2114.46 | 1298.4 | 0.703125797 | 0.00075 | 0.0357178 |
| 197 | LOC664439 | NC_007422.5:10416132-10418741 | 664439 | acyl-CoA synthetase family member 3, mitochondrial | 50.4842 | 31.0425 | 0.684143038 | 0.0005 | 0.0259129 |
| 198 | - | NW_015451782.1:48431-48717 | - | - | 2002.84 | 1234.33 | 0.697870831 | 5.00E-05 | 0.00352415 |
| 199 | LOC656992 | NC_007424.3:5204660-5208030 | 656992 | 4-aminobutyrate aminotransferase, mitochondrial-like | 67.1836 | 41.4988 | 0.682002668 | 5.00E-05 | 0.00352415 |
| 200 | - | NW_015451463.1:1816-2091 | - | - | 2040.91 | 1263.34 | 0.691534801 | 5.00E-05 | 0.00352415 |
| 201 | Nat | NC_007419.2:6607412-6617811 | 664229 | dopamine N acetyltransferase | 213.457 | 133.072 | 0.677680435 | 0.00055 | 0.0281853 |
| 202 | LOC655942 | NC_007421.3:2045203-2047987 | 655942 | protein GUCD1 | 53.3453 | 33.2693 | 0.665238548 | 0.0011 | 0.0486442 |
| 203 | LOC655491 | NC_007417.3:3288378-3296663 | 655491 | tubulin alpha 1-like protein | 77.6973 | 48.5684 | 0.666893448 | 0.00035 | 0.0194127 |
| 204 | - | NW_015451371.1:7855-8898 | - | - | 752.714 | 472.036 | 0.672067208 | 0.0008 | 0.037707 |
| 205 | LOC662761 | NC_007418.3:687991-691412 | 662761 | adenosine deaminase CECR1 | 89.3882 | 56.0948 | 0.662775088 | 0.0003 | 0.0169681 |
| 206 | LOC664154 | NC_007417.3:7763122-7780118 | 664154 | uncharacterized LOC664154 | 89.2228 | 56.2431 | 0.656390256 | 0.0006 | 0.0297494 |
| 207 | - | NC_007424.3:14489446-14491160 | - | - | 60.6251 | 38.6516 | 0.636139001 | 0.0006 | 0.0297494 |
| 208 | LOC659321 | NC_007420.3:12620445-12623447 | 659321 | tyrosine aminotransferase | 206.15 | 131.495 | 0.644737903 | 0.0005 | 0.0259129 |
| 209 | LOC100142601 | NC_007419.2:1397019-1402677 | 100142601 | phosphoribosylformylglycinamidine synthase | 42.277 | 27.1392 | 0.621019181 | 0.0011 | 0.0486442 |
| 210 | LOC660688 | NW_015451036.1:167479-175442 | 660688 | proline-rich extensin-like protein EPR1 | 27.6115 | 17.7787 | 0.607497943 | 0.0007 | 0.0338289 |
| 211 | Arr1 | NC_007420.3:3790766-3798103 | 657618 | arrestin 1 | 73.5273 | 47.3804 | 0.623346297 | 0.0005 | 0.0259129 |
| 212 | LOC661336 | NC_007422.5:3353677-3363322 | 661336 | neuroendocrine convertase 2 | 58.8818 | 37.9922 | 0.618932032 | 0.0005 | 0.0259129 |
| 213 | LOC103314006 | NC_007423.3:11127895-11132433 | 103314006 | prohormone-2 | 217.74 | 142.182 | 0.611366927 | 0.00075 | 0.0357178 |
| 214 | LOC654884 | NC_007422.5:7976218-7984456 | 654884 | abhydrolase domain-containing protein 2 | 119.821 | 78.4816 | 0.604178412 | 0.00045 | 0.0239168 |

| | | | | | | | | | |
|---|---|---|---|---|---|---|---|---|---|
| 215 | LOC655020 | NC_007422.5:4256193-4265582 | 655020 | protein phosphatase 1A | 69.9566 | 104.739 | -0.575498801 | 0.00065 | 0.0319518 |
| 216 | Toll3 | NC_007417.3:4930716-4940271 | 656158 | toll-like receptor 3 | 16.4606 | 24.6816 | -0.556631952 | 0.001 | 0.0455408 |
| 217 | LOC663635 | NC_007420.3:9273120-9279317 | 663635 | sterol regulatory element-binding protein cleavage-activating protein | 15.2285 | 23.1091 | -0.571048138 | 0.0004 | 0.0218162 |
| 218 | LOC660224 | NC_007418.3:258591-259603 | 660224 | rhythmically expressed gene 2 protein | 96.9004 | 149.441 | -0.619811142 | 0.0004 | 0.0218162 |
| 219 | LOC657042 | NC_007423.3:13439529-13441914 | 657042 | nucleolar complex protein 2 homolog | 14.6192 | 22.8659 | -0.611630181 | 0.00055 | 0.0281853 |
| 220 | LOC661892 | NC_007422.5:12270478-12278083 | 661892 | protein SOX-15 | 23.0709 | 36.1888 | -0.627578112 | 0.00085 | 0.0395751 |
| 221 | LOC658340 | NC_007418.3:53870-59915 | 658340 | UNC93-like protein MFSD11 | 58.2738 | 91.467 | -0.641544032 | 0.00075 | 0.0357178 |
| 222 | LOC663003 | NC_007424.3:11843371-11846305 | 663003 | uncharacterized LOC663003 | 12.6591 | 19.9564 | -0.617528477 | 0.0006 | 0.0297494 |
| 223 | LOC660215 | NC_007416.3:4422796-4423566 | 660215 | uncharacterized LOC660215 | 127.291 | 200.828 | -0.653706372 | 0.0002 | 0.0120247 |
| 224 | LOC659000 | NC_007418.3:5146248-5148473 | 659000 | protein SDA1 homolog | 10.3984 | 16.4573 | -0.614999118 | 0.001 | 0.0455408 |
| 225 | LOC100142105 | NC_007421.3:4278613-4281820 | 100142105 | brain protein I3 | 99.6908 | 157.785 | -0.657142759 | 0.0005 | 0.0259129 |
| 226 | Cyp346a2 | NC_007420.3:9730439-9732117 | 657412 | cytochrome P450 346A2 | 30.9659 | 49.0199 | -0.64596846 | 0.00025 | 0.014684 |
| 227 | LOC661684 | NC_007421.3:775511-777185 | 661684 | short-chain specific acyl-CoA dehydrogenase, mitochondrial | 24.5462 | 39.0937 | -0.650266855 | 0.0003 | 0.0169681 |
| 228 | LOC662326 | NC_007417.3:14939348-14943419 | 662326 | heat shock 70 kDa protein cognate 2 | 26.859 | 42.7952 | -0.652629284 | 0.0002 | 0.0120247 |
| 229 | Csp18 | NC_007422.5:13932549-13933016 | 661601 | chemosensory protein 18 | 465.515 | 741.726 | -0.670906613 | 0.00025 | 0.014684 |
| 230 | LOC660592 | NC_007418.3:10096889-10113150 | 660592 | N-acetylgalactosamine kinase | 31.6173 | 50.5195 | -0.659481229 | 5.00E-05 | 0.00352415 |
| 231 | LOC658388 | NW_015450712.1:76662-78322 | 658388 | cytochrome P450 6BR1 | 16.0028 | 25.6226 | -0.646879127 | 0.0005 | 0.0259129 |
| 232 | LOC662079 | NC_007419.2:2625995-2631595 | 662079 | hypothetical protein | 19.6107 | 31.4021 | -0.652693814 | 0.00025 | 0.014684 |
| 233 | LOC662383 | NC_007423.3:4119857-4126194 | 662383 | protein msta, isoform B | 15.1998 | 24.5617 | -0.658007785 | 0.00045 | 0.0239168 |
| 234 | LOC659207 | NC_007417.3:45734-68728 | 659207 | transferrin | 17.6007 | 28.4732 | -0.664046792 | 0.00105 | 0.0470692 |
| 235 | LOC655313 | NC_007416.3:3756168-3765231 | 655313 | semaphorin-5A | 28.2365 | 45.6881 | -0.675284267 | 0.00025 | 0.014684 |
| 236 | LOC655245 | NC_007423.3:167402-169487 | 655245 | aldehyde dehydrogenase, dimeric NADP-preferring | 36.2413 | 58.7413 | -0.681825197 | 5.00E-05 | 0.00352415 |
| 237 | LOC103313009 | NC_007417.3:1760229-1763825 | 103313009 | uncharacterized LOC103313009 | 154.96 | 251.789 | -0.696757627 | 0.00055 | 0.0281853 |
| 238 | LOC655423 | NC_007418.3:1146005-1147378 | 655423 | alpha-tocopherol transfer protein-like | 38.3678 | 62.546 | -0.690785223 | 0.0002 | 0.0120247 |
| 239 | SA; stromalin | NC_007420.3:9385414-9389924 | 655276 | cohesin subunit SA-1 | 8.52965 | 13.9946 | -0.653947903 | 5.00E-05 | 0.00352415 |
| 240 | LOC656104 | NC_007421.3:63465-71878 | 656104 | T-complex protein 1 subunit zeta | 47.4364 | 77.8406 | -0.702847114 | 0.0001 | 0.00663971 |
| 241 | LOC659785 | NC_007422.5:6522954-6527857 | 659785 | probable cleavage and polyadenylation specificity factor subunit 2 | 10.3888 | 17.0823 | -0.666962452 | 0.0002 | 0.0120247 |
| 242 | LOC103314479 | NC_007425.3:6234058-6237814 | 103314479 | uncharacterized LOC103314479 | 7.33904 | 12.1694 | -0.659236404 | 0.00035 | 0.0194127 |
| 243 | LOC664562 | NC_007422.5:9833967-9838122 | 664562 | glucosylceramidase | 41.8783 | 69.5296 | -0.717981149 | 5.00E-05 | 0.00352415 |
| 244 | LOC655815 | NC_007418.3:3583259-3591506 | 655815 | protein BTG1 | 64.3603 | 106.898 | -0.723181613 | 0.0001 | 0.00663971 |
| 245 | LOC656298 | NC_007418.3:14512305-14528095 | 656298 | annexin B9 | 71.0097 | 118.087 | -0.72575277 | 0.0009 | 0.0416491 |
| 246 | LOC103314187 | NW_015450232.1:77786-80988 | 103314187 | serine protease snake | 29.4396 | 49.0301 | -0.716846935 | 0.0008 | 0.037707 |
| 247 | LOC103312942 | NC_007420.3:4705674-4708861 | 103312942 | uncharacterized LOC103312942 | 22.5772 | 37.6704 | -0.713837292 | 0.0001 | 0.00663971 |
| 248 | LOC659195 | NC_007424.3:286609-287649 | 659195 | troponin C, isoform 1 | 315.193 | 527.319 | -0.740603866 | 5.00E-05 | 0.00352415 |
| 249 | LOC657335 | NC_007420.3:8152126-8155578 | 657335 | sulfotransferase family cytosolic 1B member 1 | 61.3575 | 102.723 | -0.734100845 | 5.00E-05 | 0.00352415 |
| 250 | ML1 | NC_007419.2:6588287-6589812 | 664531 | MD2-like 1 | 278.172 | 465.905 | -0.741974787 | 5.00E-05 | 0.00352415 |
| 251 | LOC658084 | NC_007418.3:3667009-3673954 | 658084 | palmitoyl-protein thioesterase 1 | 14.1859 | 23.9438 | -0.715948853 | 0.00015 | 0.00949185 |
| 252 | LOC663261 | NC_007424.3:12139213-12141584 | 663261 | probable 28S rRNA (cytosine-C(5))-methyltransferase | 8.46807 | 14.3689 | -0.698871633 | 5.00E-05 | 0.00352415 |
| 253 | LOC662530 | NW_015450798.1:17423-18657 | 662530 | transmembrane emp24 domain-containing protein bai | 55.0696 | 93.5047 | -0.753167303 | 5.00E-05 | 0.00352415 |
| 254 | LOC661450 | NC_007416.3:7275308-7297021 | 661450 | solute carrier organic anion transporter family member 5A1 | 9.40076 | 16.0085 | -0.709566961 | 0.0011 | 0.0486442 |
| 255 | rad50 | NC_007421.3:3442759-3447047 | 658288 | rad50 | 2.10939 | 3.59625 | -0.563825691 | 0.0011 | 0.0486442 |
| 256 | LOC655289 | NC_007422.5:1759507-1765805 | 655289 | pentatricopeptide repeat-containing protein 2, mitochondrial | 15.7703 | 26.9391 | -0.736377051 | 0.00025 | 0.014684 |
| 257 | Gr106 | NC_007420.3:8958318-8966093 | 662798 | glucose transporter | 30.0719 | 51.4642 | -0.755722844 | 0.0007 | 0.0338289 |
| 258 | LOC100142097 | NC_007423.3:11760941-11761714 | 100142097 | uncharacterized LOC100142097 | 278.576 | 478.021 | -0.776848385 | 5.00E-05 | 0.00352415 |

| | | | | | | | | | | |
|---|---|---|---|---|---|---|---|---|---|---|
| 259 | LOC655370 | NC_007421.3:3124430-3133836 | 655370 | uncharacterized LOC655370 | 14.7297 | 25.278 | -0.740364322 | 5.00E-05 | 0.00352415 |
| 260 | LOC107399196 | NW_015452199.1:1353-2623 | 107399196 | uncharacterized LOC107399196 | 46.882 | 80.7461 | -0.771666489 | 5.00E-05 | 0.00352415 |
| 261 | LOC656820 | NC_007422.5:5375003-5382661 | 656820 | C-1-tetrahydrofolate synthase, cytoplasmic | 55.3313 | 95.3973 | -0.775055972 | 5.00E-05 | 0.00352415 |
| 262 | LOC662773 | NC_007422.5:489656-490132 | 662773 | tenecin-3 | 296.209 | 514.945 | -0.795739478 | 5.00E-05 | 0.00352415 |
| 263 | LOC103314922 | NC_007420.3:7395440-7396587 | 103314922 | uncharacterized LOC103314922 | 23.4531 | 40.9041 | -0.777074036 | 0.00075 | 0.0357178 |
| 264 | LOC103313789 | NC_007423.3:3817639-3826196 | 103313789 | uncharacterized LOC103313789 | 14.0284 | 24.5514 | -0.76571092 | 5.00E-05 | 0.00352415 |
| 265 | LOC655860 | NC_007420.3:14729016-14731204 | 655860 | nucleolar GTP-binding protein 2 | 9.25317 | 16.2081 | -0.747017793 | 0.0001 | 0.00663971 |
| 266 | LOC100142246 | NC_007417.3:12105006-12111871 | 100142246 | transmembrane protease serine 9 | 36.7111 | 64.7702 | -0.802444824 | 0.00045 | 0.0239168 |
| 267 | LOC659445 | NC_007418.3:128530-136783 | 659445 | importin-7 | 23.1932 | 40.9247 | -0.793198855 | 0.0007 | 0.0338289 |
| 268 | daw; Alp23B | NW_015451971.1:37267-41548 | 658911 | dawdle | 6.97833 | 12.3159 | -0.738991239 | 0.0006 | 0.0297494 |
| 269 | LOC663832 | NC_007422.5:11420618-11426051 | 663832 | aspartic proteinase A2 | 139.137 | 245.703 | -0.815937342 | 5.00E-05 | 0.00352415 |
| 270 | LOC659989 | NC_007421.3:8060009-8076166 | 659989 | zinc carboxypeptidase | 50.6973 | 89.5994 | -0.809412561 | 0.0003 | 0.0169681 |
| 271 | LOC664209 | NC_007423.3:9067215-9069530 | 664209 | uncharacterized LOC664209 | 155.437 | 275.543 | -0.821922049 | 5.00E-05 | 0.00352415 |
| 272 | TcasZ9desA | NC_007425.3:6773285-6786677 | 658103 | Z9 acyl-CoA desaturase A | 156.491 | 277.739 | -0.823645486 | 0.00075 | 0.0357178 |
| 273 | serpin24 | NC_007423.3:5884658-5886989 | 662831 | serpin peptidase inhibitor 24 | 18.4766 | 32.9658 | -0.802340984 | 5.00E-05 | 0.00352415 |
| 274 | ML5 | NC_007422.5:6846774-6848499 | 656806 | MD2-like 5 | 129.41 | 231.391 | -0.833499696 | 5.00E-05 | 0.00352415 |
| 275 | LOC100142066 | NC_007417.3:2909684-2920265 | 100142066 | uncharacterized LOC100142066 | 9.68944 | 17.3362 | -0.77850841 | 0.0008 | 0.037707 |
| 276 | LOC658596 | NC_007422.5:8324494-8335126 | 658596 | nuclear pore membrane glycoprotein 210 | 3.43892 | 6.1997 | -0.697728085 | 5.00E-05 | 0.00352415 |
| 277 | LOC655281 | NC_007423.3:9702093-9703508 | 655281 | uncharacterized LOC655281 | 40.9712 | 73.9293 | -0.836130262 | 5.00E-05 | 0.00352415 |
| 278 | LOC663867 | NC_007422.5:13157788-13158793 | 663867 | proteasome subunit beta type-4 | 68.3952 | 123.59 | -0.844280496 | 5.00E-05 | 0.00352415 |
| 279 | LOC659791 | NC_007421.3:5616218-5655473 | 659791 | alpha-tocopherol transfer protein-like | 30.1196 | 54.4762 | -0.834045453 | 0.00025 | 0.014684 |
| 280 | LOC658566 | NC_007418.3:1393517-1395804 | 658566 | 4-coumarate--CoA ligase 1 | 43.6587 | 79.3134 | -0.846699461 | 0.0008 | 0.037707 |
| 281 | LOC103313132 | NC_007422.5:5992519-6006521 | 103313132 | uncharacterized LOC103313132 | 4.59717 | 8.36909 | -0.743211362 | 0.00015 | 0.00949185 |
| 282 | - | NC_007422.5:8836159-8837521 | - | - | 34.3424 | 62.6722 | -0.849263601 | 5.00E-05 | 0.00352415 |
| 283 | LOC658982 | NW_015451959.1:3177-5999 | 658982 | periodic tryptophan protein 2 homolog | 4.2268 | 7.73082 | -0.740189202 | 0.0006 | 0.0297494 |
| 284 | LOC656402 | NC_007416.3:105572-111276 | 656402 | glycogenin-2 | 92.1874 | 168.819 | -0.865791078 | 5.00E-05 | 0.00352415 |
| 285 | LOC661235 | NC_007418.3:18833724-18842456 | 661235 | pre-rRNA-processing protein TSR1 homolog | 8.42663 | 15.4628 | -0.804395723 | 0.00045 | 0.0239168 |
| 286 | LanA | NC_007418.3:5388902-5421238 | 661583 | laminin subunit alpha | 2.05359 | 3.7765 | -0.645447498 | 0.00075 | 0.0357178 |
| 287 | Y-1 | NC_007423.3:13098920-13102773 | 662975 | yellow-1 | 35.6633 | 65.5874 | -0.860912563 | 5.00E-05 | 0.00352415 |
| 288 | LOC663110 | NC_007424.3:4101781-4102842 | 663110 | protein BCCIP homolog | 17.0472 | 31.4301 | -0.84555845 | 0.0001 | 0.00663971 |
| 289 | LOC655179 | NC_007417.3:10821015-10824320 | 655179 | WD repeat-containing protein 36 | 2.73119 | 5.0532 | -0.698062192 | 0.0006 | 0.0297494 |
| 290 | LOC658565 | NC_007418.3:60561-75519 | 658565 | arf-GAP with Rho-GAP domain, ANK repeat and PH domain-containing protein [illegible] | 16.2619 | 30.1676 | -0.852455798 | 0.0007 | 0.0338289 |
| 291 | LOC103313795 | NC_007423.3:4191208-4197137 | 103313795 | hypothetical protein | 10.5553 | 19.876 | -0.853290591 | 0.0003 | 0.0169681 |
| 292 | P37 | NC_007418.3:11916031-11923284 | 655840 | serine protease P37 | 107.295 | 202.289 | -0.908565518 | 5.00E-05 | 0.00352415 |
| 293 | LOC657463 | NC_007416.3:28041-32308 | 657463 | sarcalumenin | 68.0665 | 128.393 | -0.905701551 | 5.00E-05 | 0.00352415 |
| 294 | Cda8 | NC_007420.3:6638235-6639855 | 662008 | chitin deacetylase 8 | 102.165 | 192.787 | -0.909518191 | 0.0004 | 0.0218162 |
| 295 | Cyp4q1 | NC_007424.3:9524439-9526264 | 659878 | cytochrome P450 monooxigenase CYP4Q1 | 26.2928 | 49.7315 | -0.894361408 | 5.00E-05 | 0.00352415 |
| 296 | LOC663981 | NC_007417.3:12412614-12414764 | 663981 | epoxide hydrolase 4 | 5.29294 | 10.0192 | -0.808213395 | 0.0002 | 0.0120247 |
| 297 | LOC103314725 | NW_015450989.1:282168-320687 | 103314725 | eIF-2-alpha kinase activator GCN1 | 4.28045 | 8.13035 | -0.790009284 | 0.00015 | 0.00949185 |
| 298 | LOC103313111 | NC_007420.3:9088174-9097602 | 103313111 | collagen alpha-2(IV) chain | 5.6274 | 10.691 | -0.818883436 | 5.00E-05 | 0.00352415 |
| 299 | LOC664288 | NC_007419.2:9234736-9243034 | 664288 | leucine--tRNA ligase, cytoplasmic | 9.63733 | 18.3311 | -0.861787658 | 0.0002 | 0.0120247 |
| 300 | LOC103312937 | NC_007420.3:4528636-4530444 | 103312937 | sodium/potassium-transporting ATPase subunit beta-2 | 10.175 | 19.4098 | -0.868987214 | 5.00E-05 | 0.00352415 |
| 301 | LOC659823 | NW_015452129.1:9-1304 | 659823 | pescadillo homolog | 10.3673 | 19.8592 | -0.875794207 | 5.00E-05 | 0.00352415 |
| 302 | LOC103312293 | NC_007418.3:4255638-4262123 | 103312293 | paramyosin | 3.87539 | 7.4255 | -0.789244674 | 0.0004 | 0.0218162 |

| | | | | | | | | | |
|---|---|---|---|---|---|---|---|---|---|
| 303 | LOC655207 | NC_007421.3:2015475-2022700 | 655207 | carboxypeptidase D | 87.7883 | 168.364 | -0.931685764 | 0.0006 | 0.0297494 |
| 304 | LOC664364 | NC_007417.3:8617585-8620167 | 664364 | SPARC | 54.3415 | 104.505 | -0.930877718 | 5.00E-05 | 0.00352415 |
| 305 | MRP | NC_007421.3:188314-195426 | 658210 | probable multidrug resistance-associated protein lethal(2)03659 | 4.32175 | 8.32368 | -0.808998752 | 5.00E-05 | 0.00352415 |
| 306 | LOC661092 | NC_007417.3:13240881-13248493 | 661092 | sodium/potassium-transporting ATPase subunit alpha | 5.26452 | 10.1432 | -0.830887715 | 5.00E-05 | 0.00352415 |
| 307 | LOC662517 | NC_007422.5:13780430-13781456 | 662517 | deoxyuridine 5'-triphosphate nucleotidohydrolase | 11.6912 | 22.5925 | -0.894499815 | 0.0002 | 0.0120247 |
| 308 | LOC656355 | NC_007420.3:14744243-14746019 | 656355 | regucalcin | 32.7036 | 63.4655 | -0.935624581 | 5.00E-05 | 0.00352415 |
| 309 | P139; P138 | NC_007422.5:6041473-6047386 | 661211 | serine protease easter | 9.55014 | 18.5341 | -0.888732644 | 0.0002 | 0.0120247 |
| 310 | LOC103313602 | NC_007422.5:4646200-4647370 | 103313602 | hypothetical protein | 11.003 | 21.3714 | -0.898260508 | 0.0001 | 0.00663971 |
| 311 | LOC659125 | NC_007420.3:1867701-1875585 | 659125 | dual specificity protein phosphatase 14 | 12.5696 | 24.4162 | -0.905370154 | 5.00E-05 | 0.00352415 |
| 312 | LOC661261 | NC_007420.3:10014458-10017089 | 661261 | major facilitator superfamily domain-containing protein 1 | 10.259 | 20.0683 | -0.903995212 | 5.00E-05 | 0.00352415 |
| 313 | LOC663579 | NC_007418.3:5980961-5982150 | 663579 | zinc transporter ZIP1 | 20.6991 | 40.6013 | -0.938993406 | 5.00E-05 | 0.00352415 |
| 314 | LOC662498 | NW_015450798.1:3368-13812 | 662498 | senecionine N-oxygenase | 61.9099 | 121.642 | -0.963094155 | 5.00E-05 | 0.00352415 |
| 315 | Z9desB | NW_015450728.1:196850-224677 | 657939 | Z9 acyl-CoA desaturase B | 73.9655 | 145.53 | -0.966897359 | 0.00015 | 0.00949185 |
| 316 | P92; P93 | NC_007422.5:6573192-6575140 | 661472 | serine protease P93 | 13.7271 | 27.1151 | -0.93287181 | 5.00E-05 | 0.00352415 |
| 317 | LOC656462 | NC_007420.3:1368354-1389670 | 656462 | pre-mRNA-splicing factor SYF1 | 4.82646 | 9.55406 | -0.857106578 | 5.00E-05 | 0.00352415 |
| 318 | LOC664474 | NC_007422.5:10079316-10081020 | 664474 | UDP-glucuronosyltransferase 2B20 | 12.8205 | 25.4082 | -0.934176159 | 5.00E-05 | 0.00352415 |
| 319 | LOC103313053 | NC_007420.3:9479790-9480417 | 103313053 | SH3 domain-binding glutamic acid-rich protein homolog | 25.7716 | 51.1543 | -0.962082841 | 0.00015 | 0.00949185 |
| 320 | LOC654859 | NC_007418.3:11533159-11608989 | 654859 | muscle M-line assembly protein unc-89 | 36.3333 | 72.3594 | -0.974518801 | 0.001 | 0.0455408 |
| 321 | LOC656773 | NC_007422.5:15123328-15125176 | 656773 | 3-ketoacyl-CoA thiolase, mitochondrial | 164.065 | 327.018 | -0.990740738 | 5.00E-05 | 0.00352415 |
| 322 | LOC660518 | NC_007419.2:4402949-4411547 | 660518 | lipid storage droplets surface-binding protein 1 | 105.807 | 212.716 | -1.000688718 | 5.00E-05 | 0.00352415 |
| 323 | LOC659539 | NC_007424.3:3531789-3552125 | 659539 | uncharacterized LOC659539 | 31.3014 | 63.0919 | -0.988545344 | 0.00105 | 0.0470692 |
| 324 | LOC103314059 | NC_007423.3:10651203-10704793 | 103314059 | kielin/chordin-like protein | 274.069 | 552.789 | -1.009542839 | 5.00E-05 | 0.00352415 |
| 325 | LOC657196 | NC_007422.5:15087077-15087895 | 657196 | protein lethal(2)essential for life | 519.507 | 1050.88 | -1.014980659 | 5.00E-05 | 0.00352415 |
| 326 | LOC660270 | NC_007420.3:7399862-7401745 | 660270 | cytochrome P450-like protein | 32.4981 | 65.783 | -0.995401634 | 0.0002 | 0.0120247 |
| 327 | LOC662842 | NC_007419.2:738655-740578 | 662842 | putative N-acetylglucosamine-6-phosphate deacetylase | 7.08987 | 14.3685 | -0.925787937 | 0.0005 | 0.0259129 |
| 328 | - | NC_007418.3:27029845-27031125 | - | - | 23.2188 | 47.4103 | -0.999186651 | 5.00E-05 | 0.00352415 |
| 329 | H4 | NC_007417.3:14188598-14191578 | 656917 | tryptase alpha/beta-1 | 44.4942 | 91.0483 | -1.016708448 | 5.00E-05 | 0.00352415 |
| 330 | LOC654972 | NC_007418.3:4222749-4228897 | 654972 | ethanolamine-phosphate cytidylyltransferase | 54.4605 | 111.825 | -1.02455425 | 5.00E-05 | 0.00352415 |
| 331 | LOC655556 | NC_007418.3:11362444-11392799 | 655556 | uncharacterized LOC655556 | 8.09174 | 16.6392 | -0.956156799 | 5.00E-05 | 0.00352415 |
| 332 | LOC100142446 | NC_007423.3:13691816-13693879 | 100142446 | kynurenine formamidase | 24.5497 | 50.669 | -1.015992611 | 5.00E-05 | 0.00352415 |
| 333 | LOC657487 | NC_007419.2:7211929-7212896 | 657487 | uncharacterized LOC657487 | 8.07788 | 16.678 | -0.961527742 | 0.00045 | 0.0239168 |
| 334 | LOC103312884 | NC_007421.3:4556300-4556688 | 103312884 | putative uncharacterized protein DDB_G0287191 | 240.839 | 498.324 | -1.045929333 | 5.00E-05 | 0.00352415 |
| 335 | LOC659994 | NC_007422.5:2209826-2213027 | 659994 | RNA-binding protein pno1 | 39.9515 | 83.4204 | -1.043675368 | 5.00E-05 | 0.00352415 |
| 336 | LOC663195 | NC_007418.3:793895-802728 | 663195 | facilitated trehalose transporter Tret1 | 21.8349 | 45.7542 | -1.033855498 | 5.00E-05 | 0.00352415 |
| 337 | LOC658429 | NC_007420.3:12101216-12111276 | 658429 | transcription factor MafA | 12.5793 | 26.3728 | -1.011333905 | 5.00E-05 | 0.00352415 |
| 338 | LOC659008 | NC_007420.3:318605-330134 | 659008 | protein takeout-like | 15.3669 | 32.4941 | -1.033125895 | 5.00E-05 | 0.00352415 |
| 339 | LOC656237 | NC_007416.3:1719129-1738731 | 656237 | lipoyltransferase 1, mitochondrial | 210.763 | 445.863 | -1.077382057 | 0.0006 | 0.0297494 |
| 340 | LOC103314203 | NC_007424.3:6233088-6233823 | 103314203 | ARL14 effector protein | 9.9784 | 21.1327 | -1.011511648 | 0.0002 | 0.0120247 |
| 341 | Cyp4q2 | NC_007424.3:9520015-9523792 | 659810 | cytochrome P450 monooxigenase CYP4Q2 | 29.3936 | 62.3018 | -1.058478957 | 0.00015 | 0.00949185 |
| 342 | LOC659452 | NC_007418.3:29326638-29443297 | 659452 | FH1/FH2 domain-containing protein 3 | 55.2307 | 117.25 | -1.072410272 | 0.0006 | 0.0297494 |
| 343 | LOC663372 | NC_007422.5:3665128-3686283 | 663372 | dystroglycan | 16.2427 | 34.5167 | -1.04251184 | 5.00E-05 | 0.00352415 |
| 344 | - | NW_015450261.1:0-2084 | - | - | 28.3137 | 60.468 | -1.068260467 | 5.00E-05 | 0.00352415 |
| 345 | LOC663877 | NC_007419.2:8872422-8873190 | 663877 | muscle-specific protein 20 | 1690.09 | 3620.88 | -1.098785306 | 0.0001 | 0.00663971 |
| 346 | LOC100141856 | NC_007418.3:15539663-15540377 | 100141856 | uncharacterized LOC100141856 | 121.382 | 260.053 | -1.092951357 | 5.00E-05 | 0.00352415 |

| | | | | | | | | | |
|---|---|---|---|---|---|---|---|---|---|
| 347 | LOC658250 | NC_007416.3:505213-512474 | 658250 | solute carrier family 41 member 1 | 4.43005 | 9.4958 | -0.950774747 | 0.0003 | 0.0169681 |
| 348 | LOC663650 | NC_007418.3:6004785-6013734 | 663650 | NFX1-type zinc finger-containing protein 1 | 11.8215 | 25.394 | -1.041644953 | 5.00E-05 | 0.00352415 |
| 349 | LOC103312157 | NW_015452154.1:11769-15913 | 103312157 | uncharacterized LOC103312157 | 14.2394 | 30.6539 | -1.054577159 | 5.00E-05 | 0.00352415 |
| 350 | LOC103312427 | NC_007418.3:17580840-17588533 | 103312427 | leucine-rich repeat-containing protein 15-like | 26.5893 | 57.9561 | -1.095532239 | 0.0011 | 0.0486442 |
| 351 | - | NC_007423.3:12240617-12241207 | - | - | 33.8376 | 74.7594 | -1.120779666 | 0.0001 | 0.00663971 |
| 352 | LOC664529 | NC_007422.5:9910444-9916586 | 664529 | patj homolog | 11.0615 | 24.5978 | -1.085610488 | 5.00E-05 | 0.00352415 |
| 353 | LOC655659 | NW_015450233.1:53802-55347 | 655659 | argininosuccinate synthase | 16.9206 | 37.85 | -1.116295658 | 5.00E-05 | 0.00352415 |
| 354 | LOC662294 | NC_007419.2:10755792-10761211 | 662294 | ATP-binding cassette sub-family G member 1 | 71.9715 | 161.29 | -1.153169091 | 5.00E-05 | 0.00352415 |
| 355 | LOC656717 | NC_007416.3:6224002-6240493 | 656717 | Down syndrome cell adhesion molecule-like protein Dscam2 | 6.01681 | 13.4979 | -1.04695674 | 5.00E-05 | 0.00352415 |
| 356 | LOC660984 | NC_007419.2:7653637-7656654 | 660984 | leucine-rich repeat-containing protein 15 | 9.43468 | 21.1871 | -1.088334752 | 5.00E-05 | 0.00352415 |
| 357 | LOC103312995 | NC_007420.3:6444194-6447050 | 103312995 | cubilin | 1.2824 | 2.92468 | -0.782023376 | 0.0005 | 0.0259129 |
| 358 | LOC103314029 | NC_007423.3:11763948-11764598 | 103314029 | uncharacterized LOC103314029 | 23.496 | 53.7601 | -1.160578893 | 5.00E-05 | 0.00352415 |
| 359 | Ypf1b; Irbp; Ku70 | NC_007422.5:11080951-11082768 | 664190 | inverted repeat-binding protein | 7.37564 | 16.8851 | -1.094486846 | 5.00E-05 | 0.00352415 |
| 360 | LOC659631 | NC_007417.3:11262009-11264896 | 659631 | adhesive plaque matrix protein | 19.8789 | 45.5555 | -1.15690591 | 5.00E-05 | 0.00352415 |
| 361 | LOC660368 | NC_007422.5:1063298-1064687 | 660368 | cathepsin L precursor | 566.947 | 1307.57 | -1.20416289 | 5.00E-05 | 0.00352415 |
| 362 | LOC656478 | NW_015450245.1:57584-72054 | 656478 | acyl-CoA Delta(11) desaturase-like | 9.9233 | 23.1562 | -1.144984753 | 0.00095 | 0.0436105 |
| 363 | LOC663738 | NC_007417.3:12312221-12313397 | 663738 | protein takeout | 12.4889 | 29.3171 | -1.168359055 | 5.00E-05 | 0.00352415 |
| 364 | VTGI | NC_007425.3:2112481-2114171 | 656735 | pancreatic triacylglycerol lipase | 9.24748 | 22.022 | -1.167743997 | 5.00E-05 | 0.00352415 |
| 365 | LOC103314140 | NC_007418.3:31227375-31227927 | 103314140 | hypothetical protein | 216.275 | 517.423 | -1.25460754 | 5.00E-05 | 0.00352415 |
| 366 | LOC660815 | NC_007418.3:1970626-2018832 | 660815 | tyrosine-protein kinase-like otk | 1.99625 | 4.78198 | -0.948405615 | 5.00E-05 | 0.00352415 |
| 367 | LOC655472 | NC_007425.3:1346023-1347021 | 655472 | protein takeout | 74.3584 | 179.316 | -1.258687178 | 5.00E-05 | 0.00352415 |
| 368 | LOC661444 | NC_007418.3:10645058-10664332 | 661444 | uncharacterized LOC661444 | 8.74035 | 21.248 | -1.191630131 | 0.0006 | 0.0297494 |
| 369 | LOC662582 | NC_007422.5:13771747-13772726 | 662582 | multiple organellar RNA editing factor 1, mitochondrial | 3.76983 | 9.21205 | -1.098262753 | 0.0009 | 0.0416491 |
| 370 | LOC659061 | NC_007417.3:14488131-14527717 | 659061 | lipid storage droplets surface-binding protein 1 | 46.5878 | 115.724 | -1.294437564 | 5.00E-05 | 0.00352415 |
| 371 | LOC663816 | NC_007424.3:13941478-13949379 | 663816 | copper homeostasis protein cutC homolog | 7.56796 | 18.8192 | -1.20987508 | 0.0006 | 0.0297494 |
| 372 | LOC663158 | NC_007422.5:9227393-9230671 | 663158 | vacuolar protein-sorting-associated protein 25 | 15.8657 | 39.5866 | -1.266911291 | 5.00E-05 | 0.00352415 |
| 373 | CYP6BQ13 | NC_007417.3:2344028-2348733 | 655498 | cytochrome P450 monooxygenase | 19.9793 | 50.1742 | -1.286450103 | 5.00E-05 | 0.00352415 |
| 374 | LOC664132 | NC_007417.3:7752330-7760165 | 664132 | uncharacterized LOC664132 | 8.11401 | 20.4123 | -1.232281912 | 5.00E-05 | 0.00352415 |
| 375 | LOC100142054 | NC_007424.3:12839739-12844719 | 100142054 | carboxypeptidase D | 7.49289 | 18.8606 | -1.225581739 | 5.00E-05 | 0.00352415 |
| 376 | LOC660437 | NC_007423.3:12667945-12668741 | 660437 | uncharacterized LOC660437 | 33.4953 | 84.4869 | -1.309303551 | 5.00E-05 | 0.00352415 |
| 377 | LOC103313299 | NC_007421.3:102795-111634 | 103313299 | uncharacterized LOC103313299 | 3.20777 | 8.12289 | -1.116435074 | 5.00E-05 | 0.00352415 |
| 378 | LOC100141741 | NC_007422.5:10131306-10131850 | 100141741 | endo-1,4-beta-xylanase A | 326.63 | 829.259 | -1.341493991 | 5.00E-05 | 0.00352415 |
| 379 | LOC103312195 | NC_007417.3:14784008-14786298 | 103312195 | transient receptor potential cation channel protein painless | 14.2272 | 36.1335 | -1.286070621 | 5.00E-05 | 0.00352415 |
| 380 | LOC663822 | NC_007422.5:13195554-13225411 | 663822 | papilin | 0.503907 | 1.28181 | -0.601463312 | 5.00E-05 | 0.00352415 |
| 381 | ApoLI-II | NC_007421.3:4420485-4439966 | 663181 | apolipophorins | 192.196 | 492.282 | -1.35234742 | 5.00E-05 | 0.00352415 |
| 382 | LOC656825 | NC_007417.3:4960193-4966514 | 656825 | peroxiredoxin-6 | 37.713 | 97.5877 | -1.348589553 | 5.00E-05 | 0.00352415 |
| 383 | LOC659078 | NC_007418.3:5148582-5158362 | 659078 | major facilitator superfamily domain-containing protein 6 | 8.68528 | 22.7313 | -1.292925471 | 0.00095 | 0.0436105 |
| 384 | LOC656248 | NC_007418.3:1193596-1206397 | 656248 | glycerol kinase | 10.22 | 26.834 | -1.310775576 | 0.0006 | 0.0297494 |
| 385 | LOC659396 | NC_007422.5:14371132-14404279 | 659396 | unc-112-related protein | 12.0405 | 31.6773 | -1.325289597 | 0.0005 | 0.0259129 |
| 386 | LOC103314693 | NC_007418.3:10852617-10887120 | 103314693 | N-myc protein | 8.51253 | 22.4566 | -1.302092908 | 5.00E-05 | 0.00352415 |
| 387 | H2 | NC_007417.3:14191742-14196653 | 657085 | serine protease H2 | 81.6972 | 216.999 | -1.398411129 | 5.00E-05 | 0.00352415 |
| 388 | LOC655021 | NC_007425.3:121815-283364 | 655021 | alpha-tocopherol transfer protein-like | 9.95102 | 26.5315 | -1.330017963 | 5.00E-05 | 0.00352415 |
| 389 | LOC103313284 | NC_007420.3:14473326-14486708 | 103313284 | sperm-tail PG-rich repeat-containing protein 2-like | 1.08398 | 2.89341 | -0.901692845 | 0.00035 | 0.0194127 |
| 390 | LOC103313185 | NC_007420.3:11559385-11562424 | 103313185 | transient receptor potential cation channel protein painless | 3.05371 | 8.15128 | -1.174730666 | 5.00E-05 | 0.00352415 |

| | | | | | | | | | |
|---|---|---|---|---|---|---|---|---|---|
| 391 | LOC103314736 | NC_007424.3:9708124-9710871 | 103314736 | uncharacterized LOC103314736 | 6.94336 | 18.7103 | -1.311128441 | 5.00E-05 | 0.00352415 |
| 392 | LOC100141910 | NC_007416.3:1031484-1032355 | 100141910 | uncharacterized LOC100141910 | 8.01286 | 21.6362 | -1.328574903 | 0.0003 | 0.0169681 |
| 393 | Fz2 | NC_007418.3:4400667-4448572 | 656499 | frizzled 2 | 5.49526 | 14.8874 | -1.290423863 | 0.00045 | 0.0239168 |
| 394 | LOC103313518 | NC_007422.5:485741-486808 | 103313518 | uncharacterized LOC103313518 | 14.2983 | 38.871 | -1.381968445 | 5.00E-05 | 0.00352415 |
| 395 | LOC658706 | NC_007418.3:19517182-19541777 | 658706 | tolloid-like protein 1 | 9.82519 | 26.796 | -1.360484939 | 5.00E-05 | 0.00352415 |
| 396 | LOC660692 | NC_007416.3:7235947-7240321 | 660692 | transketolase-like protein 2 | 168.19 | 463.358 | -1.45659319 | 5.00E-05 | 0.00352415 |
| 397 | LOC664291 | NC_007417.3:10691814-10694011 | 664291 | protein spartin | 3.00823 | 8.58332 | -1.257560247 | 0.0007 | 0.0338289 |
| 398 | LOC661102 | NC_007423.3:7018361-7021997 | 661102 | alpha-esterase like protein E1 | 1.1271 | 3.25283 | -0.999535327 | 5.00E-05 | 0.00352415 |
| 399 | LOC656039 | NC_007424.3:12446086-12447094 | 656039 | tetraspanin-8 | 66.5125 | 192.213 | -1.516965618 | 5.00E-05 | 0.00352415 |
| 400 | LOC654958 | NC_007421.3:1029832-1041600 | 654958 | cellular FABP-like protein | 166.78 | 489.233 | -1.546896851 | 0.00105 | 0.0470692 |
| 401 | LOC659318 | NC_007420.3:8350519-8364848 | 659318 | uncharacterized LOC659318 | 35.3372 | 103.705 | -1.526811177 | 5.00E-05 | 0.00352415 |
| 402 | LOC656405 | NC_007417.3:3308379-3309763 | 656405 | venom protease | 8.51499 | 25.1128 | -1.456483117 | 0.00015 | 0.00949185 |
| 403 | LOC103314028 | NC_007423.3:11758818-11759722 | 103314028 | hypothetical protein | 3.59869 | 10.6778 | -1.344473655 | 5.00E-05 | 0.00352415 |
| 404 | LOC103312583 | NC_007419.2:1728589-1756322 | 103312583 | uncharacterized LOC103312583 | 18.8582 | 56.1503 | -1.525026213 | 5.00E-05 | 0.00352415 |
| 405 | LOC659265 | NC_007424.3:7326977-7351458 | 659265 | phosphoinositide 3-kinase regulatory subunit 4 | 3.65907 | 10.9193 | -1.3551856 | 5.00E-05 | 0.00352415 |
| 406 | LOC655389 | NC_007425.3:4332332-4345941 | 655389 | neutral alpha-glucosidase C | 44.1687 | 132.022 | -1.55826957 | 5.00E-05 | 0.00352415 |
| 407 | CYP6BR2; CYP6BR3 | NW_015450712.1:60752-76536 | 658247 | uncharacterized LOC658247 | 8.60009 | 25.7238 | -1.477005331 | 5.00E-05 | 0.00352415 |
| 408 | LOC103313084 | NC_007420.3:8150224-8151889 | 103313084 | stromelysin-1-like | 1.17838 | 3.59997 | -1.07836881 | 0.00045 | 0.0239168 |
| 409 | LOC107397720 | NC_007419.2:9572226-9590168 | 107397720 | 4-coumarate--CoA ligase 2-like | 19.8825 | 60.8507 | -1.566495485 | 5.00E-05 | 0.00352415 |
| 410 | GLEAN_12323 | NC_007424.3:4850296-4851085 | 655079 | uncharacterized protein LOC655079 | 5.38533 | 16.6719 | -1.468624073 | 5.00E-05 | 0.00352415 |
| 411 | LOC658487 | NC_007417.3:13021892-13025659 | 658487 | zinc finger protein GLIS2 homolog | 27.8875 | 86.3857 | -1.596951858 | 5.00E-05 | 0.00352415 |
| 412 | LOC664182 | NC_007422.5:11082944-11083973 | 664182 | exosome complex component MTR3 | 4.11158 | 12.8908 | -1.442288484 | 5.00E-05 | 0.00352415 |
| 413 | LOC661049 | NC_007420.3:10003666-10012997 | 661049 | beta-lactamase-like protein 2 homolog | 98.5079 | 309.884 | -1.643493397 | 5.00E-05 | 0.00352415 |
| 414 | LOC661756 | NC_007419.2:2509618-2510608 | 661756 | uncharacterized LOC661756 | 1559.75 | 4925.9 | -1.658440724 | 5.00E-05 | 0.00352415 |
| 415 | LOC107397480 | NC_007418.3:4075263-4076325 | 107397480 | uncharacterized LOC107397480 | 38.3833 | 123.176 | -1.656730457 | 5.00E-05 | 0.00352415 |
| 416 | LOC103315089 | NC_007417.3:9904557-9905076 | 103315089 | hypothetical protein | 11.1065 | 35.9131 | -1.608351061 | 5.00E-05 | 0.00352415 |
| 417 | LOC661884 | NC_007420.3:10074491-10080962 | 661884 | cilia- and flagella-associated protein 47 | 0.320949 | 1.0423 | -0.628620037 | 5.00E-05 | 0.00352415 |
| 418 | LOC664253 | NC_007423.3:9280816-9281998 | 664253 | retinol dehydrogenase 11-like | 4.08079 | 13.2653 | -1.489385346 | 5.00E-05 | 0.00352415 |
| 419 | LOC655889 | NC_007418.3:19944068-19950826 | 655889 | jouberin | 12.3982 | 40.3232 | -1.624912786 | 5.00E-05 | 0.00352415 |
| 420 | P80 | NC_007419.2:11668481-11669467 | 655599 | serine protease P80 | 216.959 | 716.252 | -1.718423309 | 5.00E-05 | 0.00352415 |
| 421 | kinesin-5C | NC_007418.3:18199240-18204036 | 656336 | kinesin 5C | 0.957241 | 3.16133 | -1.088223291 | 5.00E-05 | 0.00352415 |
| 422 | sPLA2C | NC_007420.3:9902767-9903984 | 659784 | phospholipase A2C | 8.46914 | 28.2842 | -1.628817173 | 5.00E-05 | 0.00352415 |
| 423 | LOC103313391 | NC_007421.3:3343020-3343468 | 103313391 | uncharacterized LOC103313391 | 261.497 | 874.729 | -1.738183553 | 5.00E-05 | 0.00352415 |
| 424 | LOC654983 | NC_007419.2:7071287-7072137 | 654983 | cuticle protein 63 | 6.60227 | 22.3129 | -1.61662631 | 5.00E-05 | 0.00352415 |
| 425 | LOC657081 | NC_007425.3:5474613-5478061 | 657081 | innexin inx7 | 2.43559 | 8.57454 | -1.478645299 | 5.00E-05 | 0.00352415 |
| 426 | LOC103314603 | NC_007418.3:15659344-15661900 | 103314603 | putative nuclease HARBI1 | 3.9694 | 14.2416 | -1.616870781 | 5.00E-05 | 0.00352415 |
| 427 | LOC103313539 | NC_007422.5:830025-860944 | 103313539 | uncharacterized LOC103313539 | 18.7096 | 67.1462 | -1.789734713 | 5.00E-05 | 0.00352415 |
| 428 | LOC662944 | NC_007422.5:7373905-7398504 | 662944 | sodium/hydrogen exchanger 10 | 52.6121 | 190.67 | -1.837993993 | 0.0001 | 0.00663971 |
| 429 | serpin31 | NC_007423.3:7024846-7026186 | 661214 | serpin peptidase inhibitor 31 | 9.01205 | 32.8776 | -1.758594272 | 5.00E-05 | 0.00352415 |
| 430 | CYP346B1 | NC_007420.3:9724498-9730156 | 657187 | cytochrome P450 346B1 | 10.155 | 37.2619 | -1.778218003 | 5.00E-05 | 0.00352415 |
| 431 | LOC103313512 | NC_007422.5:495848-496453 | 103313512 | holotricin-3-like | 4.43345 | 16.3598 | -1.675809885 | 5.00E-05 | 0.00352415 |
| 432 | LOC661699 | NC_007424.3:15467402-15468942 | 661699 | radical S-adenosyl methionine domain-containing protein 2 | 12.3567 | 45.6851 | -1.805398564 | 5.00E-05 | 0.00352415 |
| 433 | LOC657759 | NW_015451620.1:66054-87805 | 657759 | thymidylate synthase | 3.5668 | 13.2108 | -1.637732259 | 5.00E-05 | 0.00352415 |
| 434 | - | NW_015451451.1:748-3157 | - | - | 2.7263 | 10.1745 | -1.584394546 | 5.00E-05 | 0.00352415 |

| | | | | | | | | | |
|---|---|---|---|---|---|---|---|---|---|
| 435 | - | NC_007417.3:170837-175251 | - | - | 9.42185 | 35.3481 | -1.802268558 | 5.00E-05 | 0.00352415 |
| 436 | LOC658244 | NC_007420.3:970982-981609 | 658244 | uncharacterized LOC658244 | 1.42336 | 5.45437 | -1.413267544 | 5.00E-05 | 0.00352415 |
| 437 | LOC657670 | NC_007424.3:5685639-5714053 | 657670 | patched domain-containing protein 3 | 16.2554 | 62.6441 | -1.882978861 | 5.00E-05 | 0.00352415 |
| 438 | LOC103312302 | NC_007418.3:30184276-30184770 | 103312302 | uncharacterized LOC103312302 | 118.485 | 467.497 | -1.971210294 | 5.00E-05 | 0.00352415 |
| 439 | LOC660178 | NC_007421.3:367354-376893 | 660178 | fatty acid synthase | 0.167013 | 0.662965 | -0.510937173 | 0.0002 | 0.0120247 |
| 440 | ilr1; InR | NC_007416.3:5210343-5247750 | 661524 | insulin-like receptor | 2.38565 | 9.54539 | -1.639107707 | 0.00095 | 0.0436105 |
| 441 | Z9desB | NW_015450728.1:196850-224677 | 657939 | Z9 acyl-CoA desaturase B | 19.4274 | 77.8752 | -1.949066169 | 5.00E-05 | 0.00352415 |
| 442 | LOC655542 | NC_007423.3:7118455-7124552 | 655542 | ribosome biogenesis protein BMS1 homolog | 1.54215 | 6.18561 | -1.499061476 | 5.00E-05 | 0.00352415 |
| 443 | Cda7 | NC_007420.3:6635069-6636388 | 100126689 | chitin deacetylase 7 | 5.10141 | 20.5562 | -1.820888293 | 5.00E-05 | 0.00352415 |
| 444 | LOC655598 | NC_007419.2:9628012-9630052 | 655598 | TBC1 domain family member 19 | 0.453191 | 1.86905 | -0.981348775 | 0.0001 | 0.00663971 |
| 445 | LOC103314558 | NW_015451937.1:352665-366702 | 103314558 | uncharacterized LOC103314558 | 0.590404 | 2.45871 | -1.120840764 | 0.00015 | 0.00949185 |
| 446 | LOC659172 | NC_007420.3:5755461-5757901 | 659172 | hypothetical protein | 0.901662 | 3.86246 | -1.354425537 | 0.0007 | 0.0338289 |
| 447 | LOC656478 | NW_015450245.1:57584-72054 | 656478 | acyl-CoA Delta(11) desaturase-like | 10.6022 | 45.7477 | -2.010496992 | 5.00E-05 | 0.00352415 |
| 448 | LOC658435 | NC_007421.3:3462031-3465600 | 658435 | cytosolic 10-formyltetrahydrofolate dehydrogenase | 3.57965 | 15.4737 | -1.846855372 | 5.00E-05 | 0.00352415 |
| 449 | LOC103313827 | NC_007422.5:14515184-14556050 | 103313827 | monocarboxylate transporter 7 | 7.62668 | 33.5906 | -2.003502692 | 0.00015 | 0.00949185 |
| 450 | LOC662305 | NC_007422.5:13796663-13799189 | 662305 | protein DEK | 5.00793 | 22.4606 | -1.965300002 | 5.00E-05 | 0.00352415 |
| 451 | LOC103315154 | NC_007417.3:5901445-5903598 | 103315154 | leucine-rich repeat-containing protein let-4-like | 0.393696 | 1.78656 | -0.99956931 | 0.0002 | 0.0120247 |
| 452 | LOC660364 | NC_007421.3:5683714-5691537 | 660364 | band 7 protein AGAP004871 | 2.41607 | 11.268 | -1.844490629 | 5.00E-05 | 0.00352415 |
| 453 | LOC662610 | NC_007421.3:1565076-1569411 | 662610 | putative fatty acyl-CoA reductase CG5065 | 2.68057 | 12.5937 | -1.884937074 | 0.00015 | 0.00949185 |
| 454 | LOC103313579 | NC_007422.5:3192754-3200292 | 103313579 | receptor-type tyrosine-protein phosphatase kappa | 0.510908 | 2.55092 | -1.232777041 | 5.00E-05 | 0.00352415 |
| 455 | gag | NW_015450678.1:10650-15931 | 100141955 | ORF 1 | 0.547611 | 2.75826 | -1.280021989 | 5.00E-05 | 0.00352415 |
| 456 | LOC103314314 | NC_007424.3:13079595-13137188 | 103314314 | brachyurin | 390.767 | 2020.48 | -2.367344146 | 0.00045 | 0.0239168 |
| 457 | LOC103312247 | NC_007418.3:1081163-1087966 | 103312247 | intraflagellar transport protein 43 homolog | 1.0107 | 5.23447 | -1.632569075 | 5.00E-05 | 0.00352415 |
| 458 | LOC661074 | NC_007424.3:15596693-15598718 | 661074 | carboxylesterase 1E | 1.62205 | 8.86383 | -1.911452739 | 5.00E-05 | 0.00352415 |
| 459 | Akh2 | NC_007420.3:13480284-13480639 | 663044 | adipokinetic hormone 2 | 6.95522 | 38.17 | -2.299775395 | 5.00E-05 | 0.00352415 |
| 460 | LOC107399289 | NC_007417.3:9749344-9751437 | 107399289 | sodium channel protein Nach-like | 0.232501 | 1.30933 | -0.90588553 | 0.00085 | 0.0395751 |
| 461 | LOC107398763 | NC_007425.3:4207123-4208204 | 107398763 | uncharacterized LOC107398763 | 16.6393 | 104.424 | -2.579338122 | 5.00E-05 | 0.00352415 |
| 462 | LOC655322 | NW_015452040.1:14345-15937 | 655322 | venom carboxylesterase-6-like | 0.315282 | 1.98004 | -1.179959545 | 0.00015 | 0.00949185 |
| 463 | LOC661561 | NC_007423.3:3374630-3390831 | 661561 | annexin A7 | 9.76415 | 61.6714 | -2.541572821 | 0.0003 | 0.0169681 |
| 464 | LOC656604 | NC_007423.3:9636269-9638323 | 656604 | venom carboxylesterase-6 | 1.57375 | 11.1145 | -2.234791032 | 5.00E-05 | 0.00352415 |
| 465 | LOC656826 | NC_007425.3:2117796-2123085 | 656826 | phospholipase A1-like | 5.97957 | 42.2679 | -2.632087039 | 5.00E-05 | 0.00352415 |
| 466 | GLEAN_02953 | NC_007418.3:5837554-5838654 | 663117 | cathepsin B precursor | 5.93956 | 42.8701 | -2.660321898 | 5.00E-05 | 0.00352415 |
| 467 | LOC107397872 | NC_007419.2:12238272-12284334 | 107397872 | uncharacterized LOC107397872 | 0.13632 | 1.05615 | -0.855576345 | 0.0003 | 0.0169681 |
| 468 | LOC660377 | NC_007423.3:10431640-10433201 | 660377 | putative protein TPRXL | 21.7418 | 174.968 | -2.951894745 | 5.00E-05 | 0.00352415 |
| 469 | Cpap1-g | NC_007422.5:11276727-11280065 | 664005 | cuticular protein analogous to peritrophins 1-G | 0.47327 | 3.903 | -1.73464291 | 5.00E-05 | 0.00352415 |
| 470 | LOC662640 | NC_007420.3:6930369-6932879 | 662640 | facilitated trehalose transporter Tret1 | 2.65113 | 22.7008 | -2.698520814 | 5.00E-05 | 0.00352415 |
| 471 | LOC660146 | NC_007420.3:7392974-7394114 | 660146 | uncharacterized LOC660146 | 11.7103 | 102.602 | -3.026981866 | 5.00E-05 | 0.00352415 |
| 472 | LOC658176 | NW_015450462.1:7116-21537 | 658176 | phosphoribosylformylglycinamidine synthase | 0.0669483 | 0.603053 | -0.587331854 | 0.0002 | 0.0120247 |
| 473 | OBP-C11; TcOBP10A | NC_007425.3:5617055-5726603 | 656161 | odorant binding protein C11 | 1085.48 | 10247.1 | -3.237622936 | 5.00E-05 | 0.00352415 |
| 474 | - | NC_007419.2:12284404-12286440 | - | - | 0.168842 | 2.05763 | -1.387333914 | 5.00E-05 | 0.00352415 |
| 475 | P23; P24 | NC_007417.3:12101023-12102083 | 662758 | serine protease P23 | 1.09272 | 13.8693 | -2.82888553 | 5.00E-05 | 0.00352415 |
| 476 | LOC657170 | NC_007417.3:14197609-14198889 | 657170 | trypsin II-P29 | 2.25799 | 34.4754 | -3.444764871 | 5.00E-05 | 0.00352415 |
| 477 | LOC100141893 | NC_007419.2:6662033-6671668 | 100141893 | uncharacterized LOC100141893 | 0.169954 | 2.85942 | -1.721932246 | 0.00025 | 0.014684 |
| 478 | LOC103312301 | NC_007418.3:30148152-30149679 | 103312301 | salivary glue protein Sgs-3-like | 4.55894 | 112.39 | -4.350339792 | 5.00E-05 | 0.00352415 |

| | | | | | | | | | |
|---|---|---|---|---|---|---|---|---|---|
| 479 | LOC657322 | NC_007418.3:4131236-4149119 | 657322 | venom carboxylesterase-6 | 2.1112 | 52.1333 | -4.094073273 | 0.0001 | 0.00663971 |
| 480 | LOC107397926 | NC_007420.3:5233008-5233767 | 107397926 | protein NPC2 homolog | 1.24216 | 33.5822 | -3.947068514 | 0.0001 | 0.00663971 |
| 481 | - | NC_007417.3:551923-552167 | - | - | 0 | 6.69672 | -2.944243764 | 5.00E-05 | 0.00352415 |
| 482 | Nplp3 | NC_007418.3:4125382-4126111 | 657788 | cuticle protein 38 | 0 | 0.947354 | -0.961515168 | 5.00E-05 | 0.00352415 |
| 483 | LOC103313848 | NC_007418.3:4126508-4126952 | 103313848 | cuticle protein 63 | 0 | 1.45399 | -1.29512937 | 5.00E-05 | 0.00352415 |
| 484 | - | NC_007419.2:3198051-3198703 | - | - | 0 | 1.21861 | -1.149656085 | 5.00E-05 | 0.00352415 |
| 485 | LOC659176 | NC_007420.3:11255780-11256299 | 659176 | endocuticle structural glycoprotein SgAbd-5 | 0 | 0.822036 | -0.865551464 | 0.001 | 0.0455408 |
| 486 | LOC661258 | NC_007420.3:6421267-6421839 | 661258 | uncharacterized LOC661258 | 0 | 0.909295 | -0.933040027 | 5.00E-05 | 0.00352415 |
| 487 | LOC659862 | NC_007421.3:9318737-9323082 | 659862 | facilitated trehalose transporter Tret1 | 0 | 0.863507 | -0.898020238 | 5.00E-05 | 0.00352415 |
| 488 | - | NC_007421.3:9911374-9911849 | - | - | 0 | 0.995359 | -0.996648336 | 5.00E-05 | 0.00352415 |
| 489 | LOC107398740 | NC_007425.3:1800702-1804977 | 107398740 | uncharacterized LOC107398740 | 0 | 5.50343 | -2.701200816 | 5.00E-05 | 0.00352415 |
| 490 | LOC656244 | NC_007425.3:6352529-6394030 | 656244 | coiled-coil domain-containing protein 186 | 0 | 2.45839 | -1.79010057 | 0.00105 | 0.0470692 |
| 491 | - | NC_007425.3:17670-18339 | - | - | 0 | 0.986724 | -0.990391464 | 5.00E-05 | 0.00352415 |
| 492 | - | NC_007425.3:1799904-1800625 | - | - | 0 | 3.58448 | -2.196758104 | 5.00E-05 | 0.00352415 |
| 493 | - | NC_007425.3:1821835-1822113 | - | - | 0 | 5.02834 | -2.591760788 | 5.00E-05 | 0.00352415 |
| 494 | - | NW_015450315.1:75893-76156 | - | - | 0 | 2.64416 | -1.865586303 | 5.00E-05 | 0.00352415 |
| 495 | - | NW_015450524.1:3960-4092 | - | - | 0 | 52.6068 | -5.744344112 | 5.00E-05 | 0.00352415 |
| 496 | LOC100142598 | NW_015450864.1:165-1246 | 100142598 | uncharacterized LOC100142598 | 0 | 1.79018 | -1.480358196 | 5.00E-05 | 0.00352415 |
| 497 | - | NW_015451540.1:63-872 | - | - | 0 | 4.81272 | -2.539213415 | 5.00E-05 | 0.00352415 |
| 498 | - | NW_015451645.1:2730-3133 | - | - | 0 | 1.1935 | -1.133234706 | 0.00025 | 0.014684 |
| 499 | - | NW_015451645.1:3193-4113 | - | - | 0 | 0.637644 | -0.71162177 | 5.00E-05 | 0.00352415 |
| 500 | - | NW_015452269.1:21-416 | - | - | 0 | 36.036 | -5.210856386 | 5.00E-05 | 0.00352415 |
| 501 | - | NC_007416.3:8469639-8469930 | - | - | 2.33447 | 0 | 1.737457469 | 0.00055 | 0.0281853 |
| 502 | LOC661960 | NC_007417.3:13341079-13411736 | 661960 | Kv channel-interacting protein 1 | 1.10321 | 0 | 1.072592907 | 0.00105 | 0.0470692 |
| 503 | LOC107397543 | NC_007418.3:11927693-11928397 | 107397543 | uncharacterized LOC107397543 | 0.660694 | 0 | 0.731786266 | 5.00E-05 | 0.00352415 |
| 504 | - | NC_007419.2:3699714-3700168 | - | - | 2.03159 | 0 | 1.600074653 | 5.00E-05 | 0.00352415 |
| 505 | LOC103313177 | NC_007420.3:11317219-11317627 | 103313177 | carbonyl reductase [NADPH | 3.22002 | 0 | 2.077249836 | 5.00E-05 | 0.00352415 |
| 506 | LOC107398167 | NC_007422.5:6049962-6051382 | 107398167 | serine protease easter-like | 0.675544 | 0 | 0.744629572 | 5.00E-05 | 0.00352415 |
| 507 | LOC107398427 | NC_007423.3:747341-747788 | 107398427 | uncharacterized LOC107398427 | 1.91857 | 0 | 1.545261671 | 5.00E-05 | 0.00352415 |
| 508 | LOC655293 | NC_007423.3:2323500-2324587 | 655293 | circumsporozoite protein | 0.570097 | 0 | 0.650853691 | 5.00E-05 | 0.00352415 |
| 509 | - | NC_007425.3:5237995-5238530 | - | - | 2.04683 | 0 | 1.607309006 | 5.00E-05 | 0.00352415 |
| 510 | LOC103312403 | NW_015450265.1:237-1091 | 103312403 | uncharacterized LOC103312403 | 2.02857 | 0 | 1.598636757 | 5.00E-05 | 0.00352415 |
| 511 | LOC103312400 | NW_015450265.1:1399-4048 | 103312400 | uncharacterized LOC103312400 | 0.728572 | 0 | 0.789580697 | 5.00E-05 | 0.00352415 |
| 512 | - | NW_015450434.1:88-653 | - | - | 1.47712 | 0 | 1.308663759 | 5.00E-05 | 0.00352415 |
| 513 | - | NW_015451025.1:44472-44704 | - | - | 13.879 | 0 | 3.895205663 | 5.00E-05 | 0.00352415 |
| 514 | - | NW_015451646.1:102584-103025 | - | - | 2.75602 | 0 | 1.909204745 | 5.00E-05 | 0.00352415 |
| 515 | - | NW_015451646.1:104058-104832 | - | - | 1.69542 | 0 | 1.430510091 | 5.00E-05 | 0.00352415 |
| 516 | - | NW_015451723.1:7990-9393 | - | - | 0.721019 | 0 | 0.783263025 | 5.00E-05 | 0.00352415 |
| 517 | - | NW_015451840.1:901-1036 | - | - | 54.374 | 0 | 5.791136835 | 5.00E-05 | 0.00352415 |
| 518 | - | NW_015451896.1:48504-48829 | - | - | 3.55401 | 0 | 2.187137459 | 5.00E-05 | 0.00352415 |
