# Supplementary material for: Transcriptomic comparison between beetle strains selected for short and long durations of death feigning
Source: Sci Rep. 2019 Sep 30;9:14001. doi: 10.1038/s41598-019-50440-5 (PMC6768993; doi:10.1038/s41598-019-50440-5)
Supplement: Supplementary file 3 — S3 Table [file 41598_2019_50440_MOESM3_ESM.pdf]

Title page for S3 Table

The title of manuscript: Transcriptomic comparison between beetle strains selected for short and long durations of death feigning.

The author list:
Hironobu Uchiyama
Ken Sasaki
Shogo Hinosawa
Keisuke Tanaka
Kentarou Matsumura
Shunsuke Yajima
Takahisa Miyatake

S3 Table. Primer sequences for qPCR analyses.

Gene	Primer	Primer sequence	Note
Reference genes			
Tcact	*Tc-act-Fw*	TCCATCATGAAGTGCGATGT	actin (Act)
	Tc-act-Rv	CACCGATCCAGACGGAGTAT	
Tctbpaf	*Tc-tbpaf-Fw*	GAAAGCGTGCTTTAGCCAAG	tbp-association factor (Tbpaf)
	Tc-tbpaf-Rv	GGTGCTTACGCCAGTGGTAT	
Target genes			
Tctat	*Tc-tat-Fw*	AAAGCCGAAAAGTGGGAAGT	tyrosine aminotransferase (Tat)
	Tc-tat-Rv	GGGTTTCAAGTTGCCGTAAA	
Tchpd	*Tc-hpd2-Fw*	TTTGATGGCTAGAGGGGTTG	4-hydroxyphenylpyruvate dioxygenase
	Tc-hpd2-Rv	CAGCAAATACCCCTTGTCGT	(Hpd)
Tcddc	*Tc-ddc3-Fw*	CGTGAGGCTGGCCTTATTCCATTT	DOPA decarboxylase (Ddc), designed
	Tc-ddc3-Rv	GTGCAGCCAGATGTTGTTCGAGTT	by Arakane et al. (2009)
Tcnat	*Tc-nat-Fw*	AACTGGGCTGCGGTATAATG	N-acetyltransferase (Nat)
	Tc-nat-Rv	GCCTTGTGGCACGATTATTT	
